# Trophozoite fitness dictates the intestinal epithelial cell response to *Giardia intestinalis* infection

**DOI:** 10.1371/journal.ppat.1011372

**Published:** 2023-05-04

**Authors:** Jana Grüttner, Jorik M. van Rijn, Petra Geiser, Alexandra Florbrant, Dominic-Luc Webb, Per M. Hellström, Magnus Sundbom, Mikael E. Sellin, Staffan G. Svärd

**Affiliations:** 1 Department of Cell and Molecular Biology, Uppsala University, Uppsala, Sweden; 2 Department of Medical Biochemistry and Microbiology, Uppsala University, Uppsala, Sweden; 3 Department of Medical Sciences, Gastroenterology and Hepatology Unit, Uppsala University, Uppsala, Sweden; 4 Department of Surgical Sciences, Uppsala University, Uppsala, Sweden; 5 Science for Life Laboratory, Uppsala University, Uppsala, Sweden; University of Washington, UNITED STATES

## Abstract

*Giardia intestinalis* is a non-invasive, protozoan parasite infecting the upper small intestine of most mammals. Symptomatic infections cause the diarrhoeal disease giardiasis in humans and animals, but at least half of the infections are asymptomatic. However, the molecular underpinnings of these different outcomes of the infection are still poorly defined. Here, we studied the early transcriptional response to *G*. *intestinalis trophozoites*, the disease-causing life-cycle stage, in human enteroid-derived, 2-dimensional intestinal epithelial cell (IEC) monolayers. Trophozoites preconditioned in media that maximise parasite fitness triggered only neglectable inflammatory transcription in the IECs during the first hours of co-incubation. By sharp contrast, “non-fit” or lysed trophozoites induced a vigorous IEC transcriptional response, including high up-regulation of many inflammatory cytokines and chemokines. Furthermore, “fit” trophozoites could even suppress the stimulatory effect of lysed trophozoites in mixed infections, suggesting active *G*. *intestinalis* suppression of the IEC response. By dual-species RNA-sequencing, we defined the IEC and *G*. *intestinalis* gene expression programs associated with these differential outcomes of the infection. Taken together, our results inform on how *G*. *intestinalis* infection can lead to such highly variable effects on the host, and pinpoints trophozoite fitness as a key determinant of the IEC response to this common parasite.

## Author summary

Diarrhoeal infectious diseases are still a major problem worldwide, each year killing nearly 800,000 children. These infections are caused by a variety of pathogenic bacteria, viruses, fungi and protozoa. The protozoan parasite *Giardia intestinalis* is the most common eukaryotic intestinal pathogen found in humans. In contrast to most bacteria and viruses that cause diarrhoea, *Giardia* parasites elicit a highly variable clinical picture and often do not cause pronounced inflammation in the intestine of infected patients. Here we show, by using human intestinal epithelial cells derived from adult intestinal stem cells, that “fit” *Giardia* parasites can actively suppress epithelial inflammatory signalling, while their “non-fit” counterparts instead induce inflammatory responses. These two faces of the parasite are associated with specific gene expression programs and may explain the earlier observed high variability in outcome of a *Giardia* infection, and its modulatory effect on infection by other intestinal pathogens.

## Introduction

*Giardia intestinalis* (also known as *G*. *lamblia* or *G*. *duodenalis*) is an intestinal protozoan parasite causing the diarrhoeal disease giardiasis in a variety of animal species [1,2]. ~180 million symptomatic human infections are estimated to occur worldwide every year [3], with the number of asymptomatic infections predicted to be even higher [4]. *Giardia intestinalis*, hereafter referred to as *G*. *intestinalis*, consists of eight different genetically heterogeneous groups or genotypes, which are classified as assemblages A-H. However, only assemblages A and B infect humans [5]. *G*. *intestinalis* infections occur primarily in children under five years of age, residing in low income countries with poor sanitation [4,6,7]. Multi-site, longitudinal birth cohort studies in low resource settings show that most children are infected before the age of two and that the infections last for extended time periods [8,9].

*G*. *intestinalis* has two main life cycle stages; the disease-causing trophozoite stage that colonises the upper small intestine and the cyst stage that is shed into the environment and fuels transmission. Trophozoites are non-invasive, but instead attach tightly to the apical surface of intestinal epithelial cells (IECs) [2]. *G*. *intestinalis* infection can cause a remarkably broad spectrum of clinical outcomes, ranging from asymptomatic carriage to acute or chronic infections [10–12]. Most infections are self-limiting but recurring *G*. *intestinalis* infections can result in malnutrition and stunting in children [4,8,13]. Common giardiasis symptoms are watery diarrhoea, bloating, epigastric pain, vomiting and nausea [2]. Acute giardiasis can also cause intestinal barrier dysfunction, including disruption of the microbiome, the mucus layer, cell junctions and inhibition of brush border enzymes [14]. The parasite may also cause long lasting effects, such as post-infectious irritable bowel syndrome and chronic fatigue syndrome [15,16]. The pathology includes a variable, but typically low, degree of mucosal inflammation, and *G*. *intestinalis* infections in humans rarely cause extensive immune cell infiltration in the infected tissue [17]. Moreover, experimental infections in mice linked *G*. *intestinalis* infections to protection against diarrhoea caused by other enteropathogens, potentially via unknown immunomodulatory mechanism(s) [14,18,19]. The molecular basis for the variable intestinal inflammatory response and the disparate clinical manifestations of *G*. *intestinalis* infection remains poorly defined.

*In vitro* cell culture systems have been extensively used to model host—parasite interactions for *G*. *intestinalis* and other pathogens. Monolayer cultures of colorectal adenocarcinoma cell lines (e.g. Caco-2 and HCT-8) constitute the most commonly used approach [20–27]. However, the reported responses of these transformed cancer cell lines to *G*. *intestinalis* infection, e.g. expression of chemokines and induction of apoptosis, have varied between studies [20,28–31]. Recently, the use of non-transformed IEC infection models in the form of human enteroids have also been reported [32,33]. Enteroids derive from epithelial adult stem cells (ASCs), and have the potential to mimic intact primary tissue better than transformed cell lines. This since they i) can be differentiated into multiple IEC lineages, while ii) retaining genomic integrity and iii) physiologically intact signalling pathways [34–37]. Thus, the use of enteroids as a model infection system has great potential to improve our understanding of host-pathogen interactions [38–42]. Still, the enteroid immune response to *G*. *intestinalis* infection remains the subject of only a single recent study [32], which revealed IEC barrier breakdown linked to cyclic AMP/protein kinase A signalling during mature stages of the infection. Notably, the authors could by RNA sequencing (RNA-seq) not detect any IEC immune response during the early infection stage (first ~1.5 h), which stands in contrast to previous works in colorectal adenocarcinoma cell line cultures [20,22]. The reasons for these discrepancies are unclear.

In this study, we have systematically probed the early transcriptional response to *G*. *intestinalis* in enteroid-derived epithelia. Jejunal enteroids from healthy human donors were used to establish 2-dimensional (2D) IEC monolayers atop transwell inserts, which were subsequently infected with *G*. *intestinalis* assemblage A trophozoites under a variety of experimental conditions. We found that trophozoites preconditioned in media that maximise parasite fitness triggered only minimal inflammatory transcription in the IECs whereas “non-fit” trophozoites induced a strong IEC response. “Fit” trophozoites could even suppress the stimulatory effect of “non-fit” trophozoites in mixed infections, suggesting active *G*. *intestinalis* immunomodulation. We also defined the IEC and *G*. *intestinalis* gene expression programs that underlie these differential infection outcomes. As such, our study begins to explain how *G*. *intestinalis* infection can lead to highly variable IEC responses, and highlights that the precise *in vitro* infection experimental condition matters greatly when studying the host response to this parasite.

## Results

### A human enteroid infection model to study early intestinal epithelial responses to *Giardia intestinalis*

To establish a human intestinal epithelial model for studying the early response of non-transformed intestinal epithelia to *Giardia* trophozoites, we evaluated 3D enteroid infections by microinjection, as well as apical infections of enteroid-derived 2D monolayers. The 3D enteroid model was evaluated by microinjecting *G*. *intestinalis* isolate WBC6 trophozoites, constitutively expressing the fluorescent protein mNeonGreen [33], into the lumen of Matrigel-embedded enteroids (S1A–S1F Fig). By time-lapse microscopy, we could detect mNeonGreen-positive trophozoites swimming in the lumen or attached to the IECs, particularly in the bottom plane (S1D–S1F Fig). This setup allowed visualization of infection progression at high resolution and frame rates over at least ~7–12 h, after which the enteroids began to deform (S1D Fig). Several factors, however, made this model less suitable for studies of epithelial responses at a reasonable throughput; the microinjections are time consuming, the *G*. *intestinalis* trophozoites can clog the needle during the microinjection process (S1A–S1C Fig), and the limited scalability makes this setup poorly compatible with quantitative RT-PCR and other bulk readouts.

**Fig 1:**
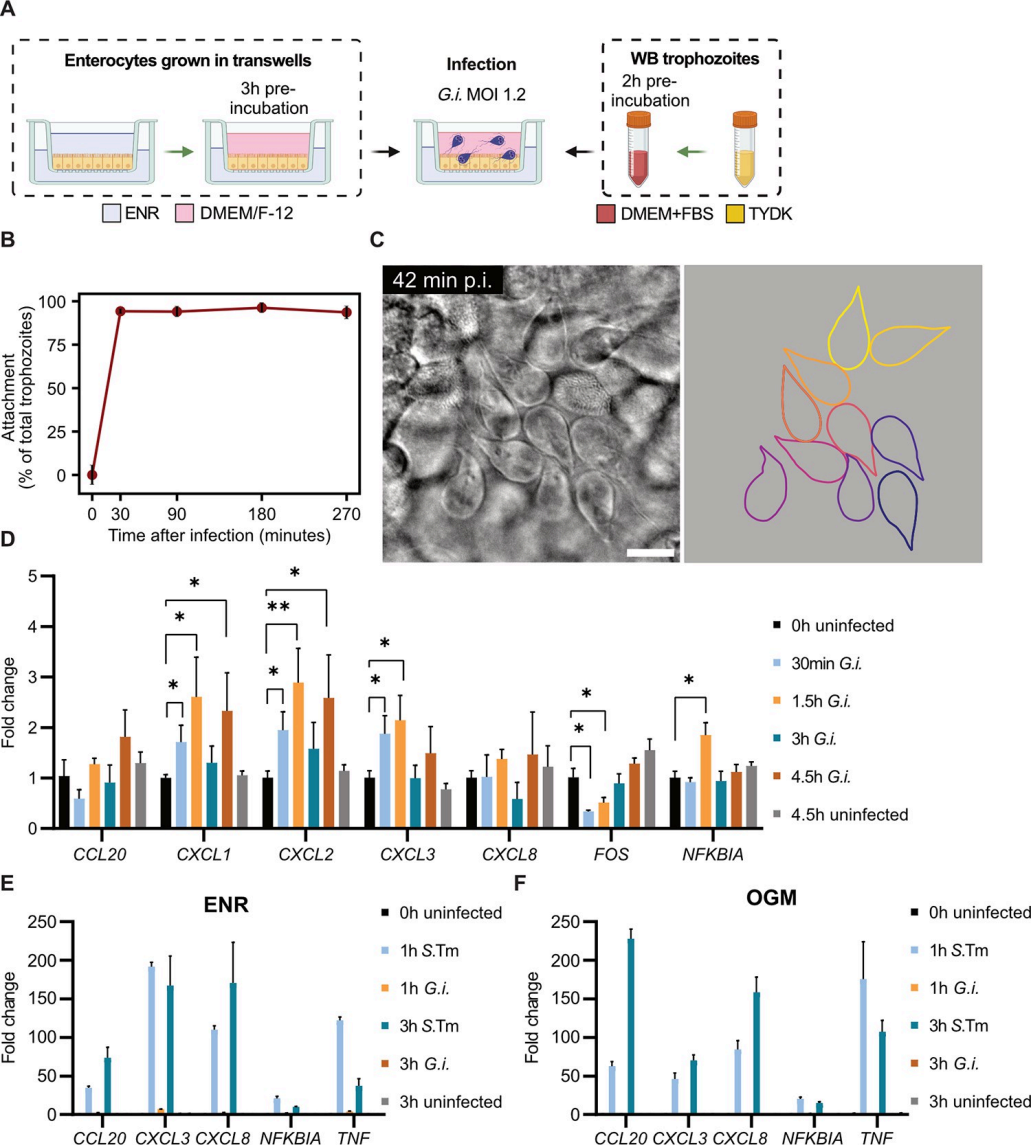
Validation of human enteroid-derived 2D intestinal epithelial monolayer infections with *Giardia intestinalis* trophozoites. (A) Schematic representation showing the jejunal enteroid-derived IEC monolayer infection model with *G*. *intestinalis* trophozoites. Created with BioRender.com. (B) *G*. *intestinalis* trophozoite attachment to IECs over the course of the monolayer infection. (C) High-definition DIC microscopy image of *G*. *intestinalis* attachment to IECs at 42 min p.i. (left panel) and illustration of the trophozoites’ outline (right panel). Scale bar = 10 μm. (D) qPCR of host cell response genes after 30 min, 1.5 h, 3 h and 4.5 h post *G*. *intestinalis* infection, including 0h and 4.5 h uninfected controls (biological replicates: n = 3 uninfected control samples, n = 4 infection samples ± SD). Fold change values of all samples were calculated by comparing to the 0h uninfected control. Statistical significance was determined using Welchs’ t-test with Holm-type corrections for multiple testing. *p < 0.05, **p < 0.01. (E) qPCR of host cell response genes in differentiated (ENR) IEC monolayers infected with *Salmonella* Typhimurium (MOI10), or *G*. *intestinalis* trophozoites (MOI1.2) after 1 h and 3 h p.i., including 0 h and 3 h uninfected controls (biological replicates: n = 3, except n = 2 for 1 h *G*. *intestinalis* infection ± SD). Fold change values of all samples were calculated by comparing to the 0 h uninfected control. (F) qPCR of host cell response genes in undifferentiated (OGM) IEC monolayers infected with *Salmonella* Typhimurium (MOI10), or *G*. *intestinalis* trophozoites (MOI1.6) after 1 h and 3 h p.i., including 0 h and 3 h uninfected controls (biological replicates n = 4 uninfected control samples, n = 3 infection samples ± SD). Fold change values of all samples were calculated by comparing to the 0 h uninfected control. *G*.*i*., *Giardia intestinalis*; *S*.Tm, *Salmonella* Typhimurium; ENR, enterocyte differentiation media; OGM, human IntestiCult organoid growth media; TYDK, *G*. *intestinalis* growth media.

For these reasons, we turned to enteroid-derived 2D monolayers, grown under stemness-maintaining conditions (complete Intesticult “OGM” medium) atop transwell inserts, and differentiated into a polarized enterocyte phenotype (“ENR” medium; see methods for details; [34]). We verified the monolayer barrier integrity by transepithelial electric resistance (TEER) measurements during the growth and differentiation of the enteroid-derived IEC monolayers (S2A Fig). The 2D monolayers were infected apically with wild-type *G*. *intestinalis* trophozoites (MOI 1.2) for up to 4.5 h post infection (p.i.) (Fig 1A), with DMEM/F-12 as the apical infection medium and ENR differentiation medium in the basal compartment. The monolayers supported swift and stable trophozoite attachment to IECs throughout the duration of the infection (Fig 1B, S1 Video). Using high-definition differential interference contrast (DIC) imaging, a method we recently introduced [33], the trophozoites were observed to attach to the epithelial surface in dense clusters (Fig 1C). Surprisingly, *G*. *intestinalis* infection elicited only negligible (~0-3-fold) induction of transcripts encoding cytokines and other epithelial defence factors, previously shown to be highly upregulated in transformed intestinal epithelial cell lines [20,22] (Fig 1D). We tested if the human enteroid-derived monolayers in principle could produce a strong transcriptional response, by conducting parallel infections with *Salmonella* Typhimurium SL1344 (*S*.Tm) (MOI10) (Fig 1E). Indeed, *S*.Tm infection triggered a vigorous upregulation (~20-190-fold) of *CCL20*, *CXCL3*, *CXCL8*, *NFKBIA*, and *TNF* transcripts at 1-3h p.i, while *G*. *intestinalis* trophozoites did not. Similar results were also obtained when the infections were repeated in undifferentiated monolayers (kept in the OGM medium) (Fig 1F), hence excluding that the IEC differentiation state would result in non-responsiveness to *G*. *intestinalis*. Enteroid responses to microbial stimuli have recently been shown to be altered by the composition of the infection medium [43]. We therefore next tested if the transcriptional response to *G*. *intestinalis* could be enhanced by using DMEM/F-12 in both the apical and basal compartment during the infection step (S2B Fig). This condition again permitted efficient *G*. *intestinalis* trophozoite attachment to the apical IEC surface (S2C Fig), but only minimal induction of inflammatory transcripts was observed also in this setup (S2D Fig).

In summary, *G*. *intestinalis* trophozoite infection of human enteroid-derived IEC monolayers, grown under several culture conditions, elicits no, or only a weak, inflammatory transcriptional response during the first 4.5 h hours of the interaction. These findings appear partially at odds with previous studies using slightly different protocols combined with colorectal adenocarcinoma cell lines (e.g. differentiated Caco-2 cells), or other cell models, to assay *G*. *intestinalis*–host cell interactions [20,22,29,44–46].

### “Non-fit” *Giardia intestinalis* trophozoites elicit a robust transcriptional response in human enteroid-derived epithelia

**Fig 2:**
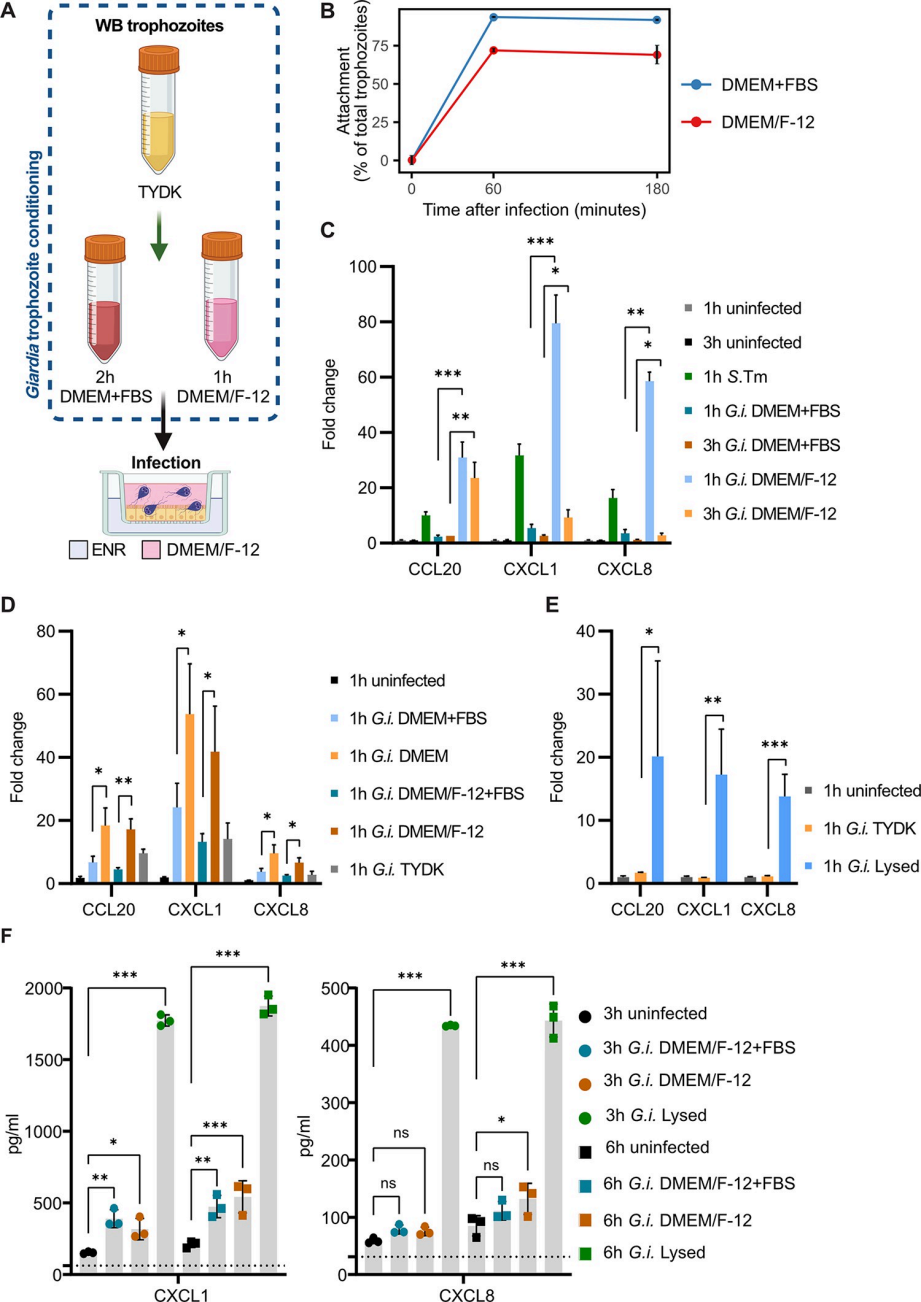
Fitness of *Giardia intestinalis* trophozoites determines the human IEC immune response. (A) Schematic representation showing *G*. *intestinalis* trophozoite media preconditioning before the infection. Created with BioRender.com. (B) *G*. *intestinalis* trophozoite attachment to IECs over the course of the infection. (C) qPCR of the indicated chemokine mRNA expression levels in IECs during infection with preconditioned *G*. *intestinalis* trophozoites (1 h and 3 h p.i.), or *Salmonella* Typhimurium (1 h p.i.) (n = 3 biological replicates ± SD). Fold change values were calculated by comparing to the respective infection time point control (1 h and 3 h uninfected, respectively). (D) qPCR of chemokine mRNA expression levels in IECs infected for 1 h with *G*. *intestinalis* trophozoites that were preconditioned before the infection in DMEM+FBS, DMEM, DMEM/F-12+FBS, or DMEM/F-12 (n = 3 biological replicates ± SD). Fold change values of all samples were calculated by comparing to the 1h uninfected control. (E) qPCR of chemokine mRNA expression levels in IECs during infection with TYDK preconditioned trophozoites or lysed trophozoites (sonicated) (1h p.i.) (n = 3 biological replicates, except two biological replicates for 1 h uninfected enteroids ± SD). Fold change values of all samples were calculated by comparing to the 1h uninfected control. No statistical tests were performed if n<3. Statistical significance for all qPCRs was determined using Welchs’ t-test with Holm-type corrections for multiple testing. (F) ELISA measurements of chemokine CXCL1 and CXCL8 protein concentrations in culture media supernatants of the basal compartment of uninfected IEC monolayers and IEC monolayers infected with DMEM/F-12+FBS preconditioned trophozoites, DMEM/F-12 preconditioned trophozoites, or lysed trophozoites at MOI1.2 (3 h and 6 h p.i.) (n = 3 biological replicates). The black dotted lines indicate the detection limit (concentration of lowest standard sample). Statistical significance was determined using a one-way analysis of variance (ANOVA), followed by Bonferroni multiple comparison test. *p < 0.05, **p < 0.01, ***p < 0.001, ns = not significant. *G*.*i*., *Giardia intestinalis*; *S*.Tm, *Salmonella* Typhimurium; ENR, enterocyte differentiation media; TYDK, *G*. *intestinalis* growth media.

As *G*. *intestinalis* triggered an unexpectedly weak IEC response (Figs 1 and S2D), we further explored a diverse set of infection conditions and *G*. *intestinalis* trophozoite preconditioning procedures. In the initial setup, trophozoites were grown in TYDK (*Giardia* growth medium containing 10% bovine serum) and incubated in DMEM supplemented with FBS (DMEM+FBS) for 2h before the infection, as previously described [47] (Fig 1A). Strikingly, we found that preconditioning trophozoites instead with the apical infection media (DMEM/F-12, lacking serum; Fig 2A), for 1 h prior to the infection changed both the trophozoite phenotype and especially the epithelial response. DMEM/F-12 preconditioned trophozoites attached less to the enteroid-derived monolayers than DMEM+FBS preconditioned ones, both at 1 h and 3 h p.i. (Fig 2B). Most importantly, DMEM/F-12 preconditioned trophozoites triggered a dramatic upregulation of IEC cytokine transcripts (Fig 2C), in sharp contrast to the DMEM+FBS preconditioned inoculum. This difference was here particularly evident in the early window of analysis, i.e. at 1 h p.i. (Fig 2C). This shows that early epithelial immune response(s) can be influenced by and depends on the phenotype of the trophozoites.

Three main factors differed between the two trophozoite preconditioning procedures: presence or absence of FBS, DMEM or DMEM/F-12 as basal medium, and the time frame used (1 or 2 h). By a systematic analysis, we found that FBS supplementation to the preconditioning medium changed the trophozoite phenotype regardless if DMEM or DMEM/F-12 was used; i.e. trophozoites preconditioned without FBS elicited a potent IEC transcriptional response (Fig 2D, see S3A Fig for corroborating IEC attachment data). This was also true in enteroid-derived monolayers established from another human donor culture (S3B Fig). By contrast, changing the preincubation time between 1h and 2h had little effect (S3C Fig).

Serum deprivation has been shown to reduce the growth rate of *G*. *intestinalis* trophozoites [48–50]. We therefore examined how the preconditioning procedures used here affected *G*. *intestinalis* fitness. After 1 h of preconditioning in DMEM/F-12, the *G*. *intestinalis* trophozoites indeed grew significantly slower than in either DMEM/F-12+FBS or TYDK (S3D Fig). In accordance with the growth arrest, DMEM/F-12 preconditioned trophozoites featured elevated intracellular ATP levels, indicating that *G*. *intestinalis* catabolism is favoured over anabolism under this condition (S3E Fig). Live/dead staining with propidium iodide (PI) and flow cytometry showed that the absence of FBS did not lead to overt killing (i.e. full permeabilisation) of the trophozoites, but still to a marginally elevated PI-signal, which may be interpreted as an increased surface stickiness often noted for stressed eukaryotic cells (S3F Fig).

To directly test if *G*. *intestinalis* trophozoite fitness impacts the IEC response, we exposed enteroid-derived monolayers in parallel to either live TYDK preconditioned trophozoites (maximally “fit”) or sonicated trophozoites (lysed and thereby dead). The TYDK preconditioned trophozoites again elicited only minimal IEC cytokine transcript expression. By striking contrast, the lysed trophozoites induced a vigorous response (Fig 2E). This formally demonstrates that *G*. *intestinalis* components can stimulate a robust immunoregulatory transcriptional program in IECs, but that “fit” *G*. *intestinalis* trophozoites either evade detection, and/or actively suppress this early epithelial response.

To examine if “non-fit”/dead trophozoites also induce elevated cytokine protein levels, we analysed the concentrations of CXCL1 and CXCL8 in the media of both the apical and basal compartment of IEC monolayers infected with DMEM/F-12+FBS (“fit”) preconditioned, DMEM/F-12 preconditioned (“non-fit”), or lysed trophozoites by ELISAs (3 h and 6 h p.i.). Notably, lysed trophozoites induced markedly elevated concentrations of CXCL1 and CXCL8 both across timepoints and compartments (Figs 2F and S4). In contrast, the apical CXCL1 and CXCL8 concentrations decreased sharply from the baseline in the presence of live parasites (“fit” and “non-fit” infections) (S4 Fig). The basal compartment CXCL1 levels increased at both time points for infection with live (“fit” and to a slightly higher extent for “non-fit”) parasites, but did not come close to the levels induced by the lysed trophozoites (Fig 2F). A similar overall trend was noted for CXCL8 (Fig 2F). Therefore, we conclude that lysed trophozoites induce a potent IEC response, evident on both transcript and protein level. Live, but “non-fit” (DMEM/F-12 preconditioned), trophozoites may on the other hand affect final cytokine protein levels in more complex ways. It is known that live trophozoites secrete high levels of proteases [21,51], shown to degrade both of the here tested cytokines [52]. This could explain the drastic decrease in cytokine protein concentrations in the apical compartment, but might also impact cytokine stability in the bottom compartment, upon infection with live (“fit” or non-fit”) parasites. Focusing the analysis on the IEC transcript response allows us to exclude such potential post-translational effects of the infection.

### Active suppression of the early epithelial response by “fit” *Giardia intestinalis* trophozoites

**Fig 3:**
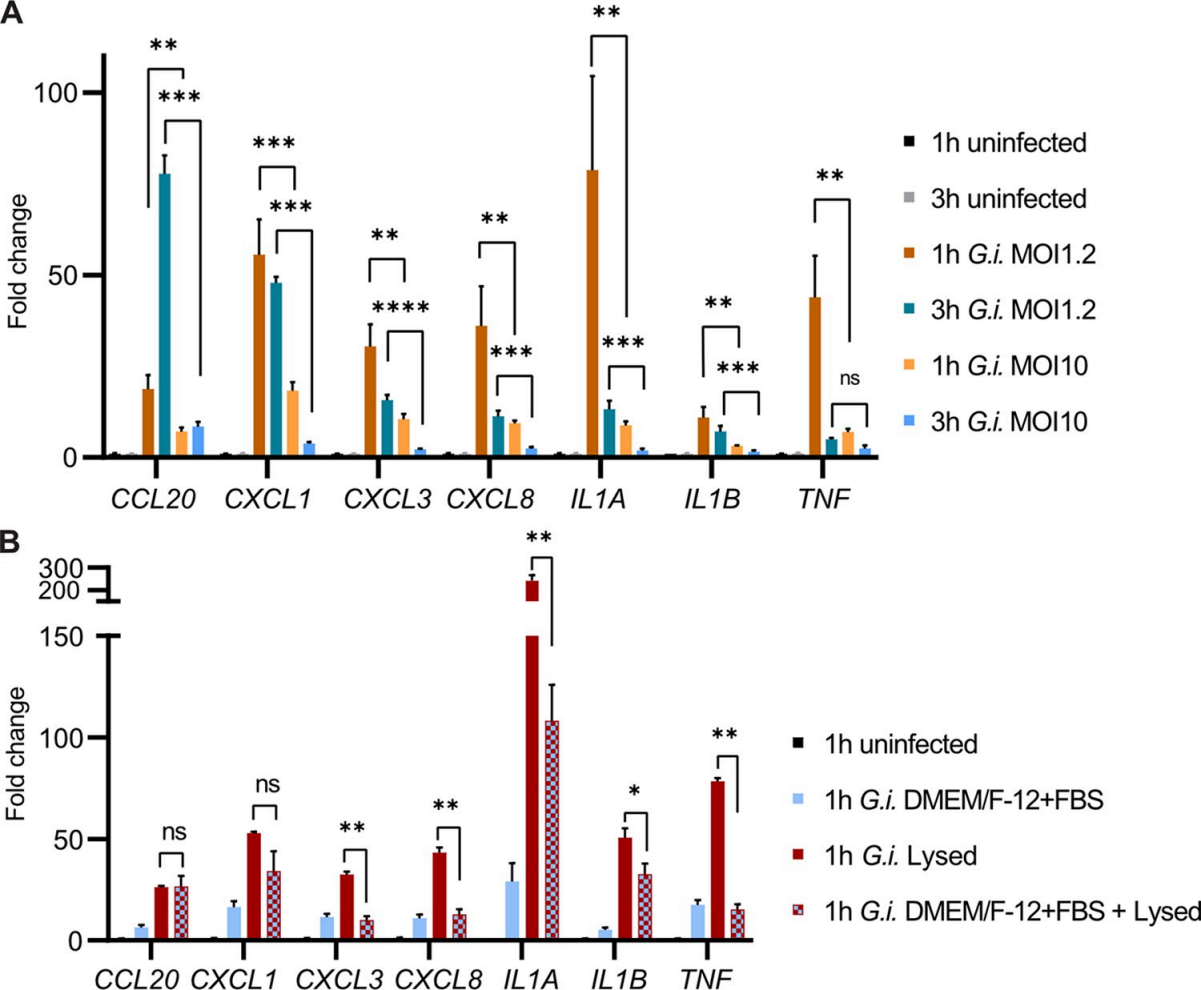
Active suppression of intestinal epithelial cell inflammatory transcription by “fit” *Giardia intestinalis* trophozoites. (A) qPCR of host response genes in IECs after MOI1.2 or MOI10 DMEM/F-12 conditioned *G*. *intestinalis* trophozoites infection (1 h and 3 h p.i.) (n = 3 biological replicates ± SD). Fold change values were calculated by comparing to the respective infection time point control (1 h and 3 h uninfected, respectively). (B) qPCR of host response genes in IECs during infection with DMEM/F-12+FBS preconditioned trophozoites, lysed trophozoites, or co-infection with both DMEM/F-12+FBS preconditioned trophozoites and lysed trophozoites (1h p.i.) (n = 3 biological replicates ± SD). All infection inoculums were added at MOI1.2. Fold change values of all samples were calculated by comparing to the 1h uninfected control. Statistical significance was determined using Welchs’ t-test with Holm-type corrections for multiple testing. *p < 0.05, **p < 0.01, ***p < 0.001, ****p < 0.0001, ns = not significant. *G*.*i*., *Giardia intestinalis*.

As shown above, “non-fit” trophozoites (preconditioned without serum) elicit significantly higher cytokine transcript upregulation than “fit” trophozoites (preconditioned with serum) (Fig 2). To address if the potent IEC-stimulatory phenotype of “non-fit” trophozoites was scalable, we increased the infection dose for DMEM/F-12 preconditioned trophozoites from MOI1.2 to MOI10. Notably, this MOI increase resulted in significantly lower induction, rather than further augmentation, of the IEC transcriptional response (Fig 3A). This shows that increasing the number of trophozoites during the epithelial infection leads to restoration of the non-stimulatory “fit” phenotype also for “non-fit” trophozoites. This observation suggests that *G*. *intestinalis* trophozoites may possess an active immunosuppressive capacity.

To further evaluate the impact of *G*. *intestinalis* components and their configuration on the magnitude of the IEC response, we next infected IEC monolayers with “fit” (i.e. DMEM/F-12+FBS preconditioned), intact but heat-inactivated (HI), or completely lysed trophozoites (S5A Fig). The lysed trophozoites caused the by far strongest cytokine transcript upregulation (S5A Fig). HI trophozoites, which still can display membrane proteins such as VSPs, but not secrete proteins, vesicles, or expose cytosolic content to the IECs, elicited significantly higher upregulation of four out of seven tested cytokine transcripts (*CCL20*, *CXCL1*, *IL1A*, and *IL1B*) compared to the “fit” trophozoites (S5A Fig), but still a markedly lower response than the lysed trophozoites (S5A Fig). This indicates that trophozoite membrane proteins cause only partial upregulation of IEC cytokine transcripts, while also other components released during *G*. *intestinalis* lysis are required for full-blown activation.

Finally, to test if “fit” trophozoites can exert active suppression of the IEC response to *G*. *intestinalis* components, we evaluated mixed infections with “fit” (again DMEM/F-12+FBS preconditioned) and lysed trophozoites. This revealed that addition of “fit” trophozoites indeed counteracted the IEC response to lysed trophozoites, as evident from significantly lowered levels of five out of seven transcripts tested, namely *CXCL3*, *CXCL8*, *IL1A*, *IL1B*, and *TNF*, and a negative trend also for *CXCL1* (Fig 3B; IEC attachment data presented in S5B Fig). Hence, our results demonstrate i) that human IECs activate swift and potent immunoregulatory transcription in response to *G*. *intestinalis* components exposed by “non-fit” or dead trophozoites, and ii) that “fit” trophozoites can actively suppress this early IEC response.

### Global RNA expression changes in intestinal epithelial cells and *Giardia intestinalis* trophozoites during early infection

We next performed dual RNA-seq analyses (simultaneous quantification of RNA transcripts of the parasite and host cells) to determine global gene expression changes in IECs exposed to “fit” (DMEM/F-12+FBS preconditioned) versus “non-fit” (DMEM/F-12 preconditioned) *G*. *intestinalis* trophozoites, as well as gene expression changes in the parasites before and during the infection. Both human and *G*. *intestinalis* read counts were readily detected in the infection samples, both at 1h and 3h p.i. (MOI ~1.2), with on average ~15% of the reads mapping to the *G*. *intestinalis* genome (S6A Fig). We also included uninfected IEC control samples, and pure *G*. *intestinalis* trophozoites harvested after the preconditioning step. As expected, >>99.9% of the reads in these samples could be confidently mapped to human and *G*. *intestinalis*, respectively (S6A Fig).

#### Transcriptomic changes in human IECs upon “fit”- versus “non-fit” trophozoite exposure

**Fig 4:**
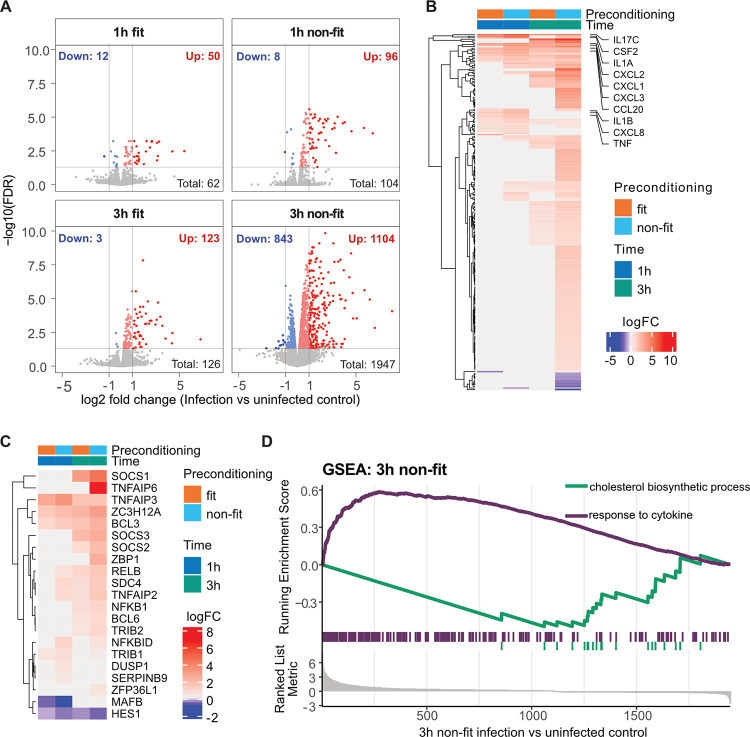
Transcriptome analysis of intestinal epithelial cells during *Giardia intestinalis* infection. (A) Volcano plots showing differentially expressed host genes (DEGs) between “fit” (DMEM/F-12+FBS) or “non-fit” (DMEM/F-12) *G*. *intestinalis* trophozoite infected IECs and uninfected control IECs at 1 h and 3 h p.i. (n = 3 biological replicates). Both infection inoculums were added at MOI1.2. Each dot represents a DEG and are coloured according to expression fold change and false discovery rate (FDR) (grey, FDR > 0.05; dark blue, FDR < 0.05 and log2FC < -1; light blue FDR < 0.05 and log2FC < 0; dark red FDR < 0.05 and log2FC > 1, light red FDR < 0.05 and log2FC > 0). (B) Heatmap showing highly up- or downregulated DEGs (log2FC < -1 or log2FC > 1) between infection and uninfected control samples at 1 and 3h p.i.. (C) Heatmap showing the log2 fold change values of immune regulating genes. Only significant values are shown. (D) Gene set enrichment assay (GSEA) plot showing negative enrichment of “cholesterol biosynthetic process” and positive enrichment of “response to cytokine” for 3h “non-fit” infection DEGs.

Principal component analysis of the top 100 variant human IEC transcripts showed a distinct clustering of all three biological replicates in each sample group, and a distinct separation of these clusters based on infection time point and condition (S6B Fig). Differential gene expression analysis revealed a moderate IEC response at 1h p.i. for both “fit” or “non-fit” *G*. *intestinalis* trophozoites, with only 62 and 104 differentially expressed genes (DEGs) (FDR < 0.05), respectively (Fig 4A, S1 Table). Very few IEC genes (12 in the “fit” and 8 in the “non-fit” trophozoite infections) were downregulated by 1h p.i. (Fig 4A, S1 Table). At 3h p.i., we could, however, observe a strong and differential IEC transcriptional response. While “fit” trophozoites caused 126 IEC genes to be differentially expressed, “non-fit” trophozoite infection produced 1947 IEC DEGs (Fig 4A, S1 Table). The spread of DEGs is illustrated in volcano plots in Fig 4A. Only 21 IEC DEGs overlapped in all conditions (S6C Fig, S2 Table). By contrast, 1780 IEC genes were differentially regulated uniquely in response to “non-fit” trophozoite infection for 3h (S6C Fig, S2 Table). Highly DEGs (above or below twofold expression difference) are illustrated in Fig 4B and the top 20 up- and downregulated DEGs are shown in S7 Fig. Both “fit” and “non-fit” *G*. *intestinalis* trophozoites elicited IEC immune response gene (encoding e.g. cytokines) upregulation, which was confirmed by Gene Ontology (GO) term analysis (Figs 4B and S8, S3 Table). However, “fit” trophozoites induced IEC cytokine transcript expression to a markedly lesser extent than “non-fit” trophozoites (Fig 4B, S1 Table). This corroborates and extends our initial analysis based on qPCR of a smaller panel of IEC transcripts, e.g. *CCL20*, *CXL1*, *2*, *3*, *8*, and *TNF* (Figs 1 and 2, compare with Fig 4B), although the magnitude and exact timing of transcript induction varied modestly between these two datasets.

We had moreover observed that “fit” *G*. *intestinalis* trophozoites can actively suppress the transcriptional response of the IECs (Fig 3). By global analysis, we here identified differential expression of several genes known to modulate immune responses, such as *TNFAIP3*, *TNFAIP6*, *SOCS1-3*, *ZBP1* and *ZC3H12A* (Fig 4C). GO term analyses further showed that terms related to signalling, oxidative stress, cell death and the apical junctional complex were enriched among upregulated DEGs in several sample groups (S8 Fig). Metabolism related terms were, on the other hand, nearly exclusively enriched among IEC genes downregulated by 3h p.i. with “non-fit” trophozoites (S8 Fig). This suggests that “non-fit” *G*. *intestinalis* trophozoites elicit strong and concomitant upregulation of immunostimulatory transcription, and downregulation of metabolic transcription, in human IECs. In further support of this notion, gene set enrichment analysis (GSEA) for the “non-fit” trophozoite infections at 3h p.i. showed that immune response related genes (e.g. included in the term “response to cytokine”), were strongly enriched among induced genes (Fig 4D), while e.g. “cholesterol biosynthetic process” related genes were enriched in the downregulated set (Fig 4D, S3 Table).

#### Transcriptomic changes in *G*. *intestinalis* trophozoites after differential preconditioning and during IEC infection

**Fig 5:**
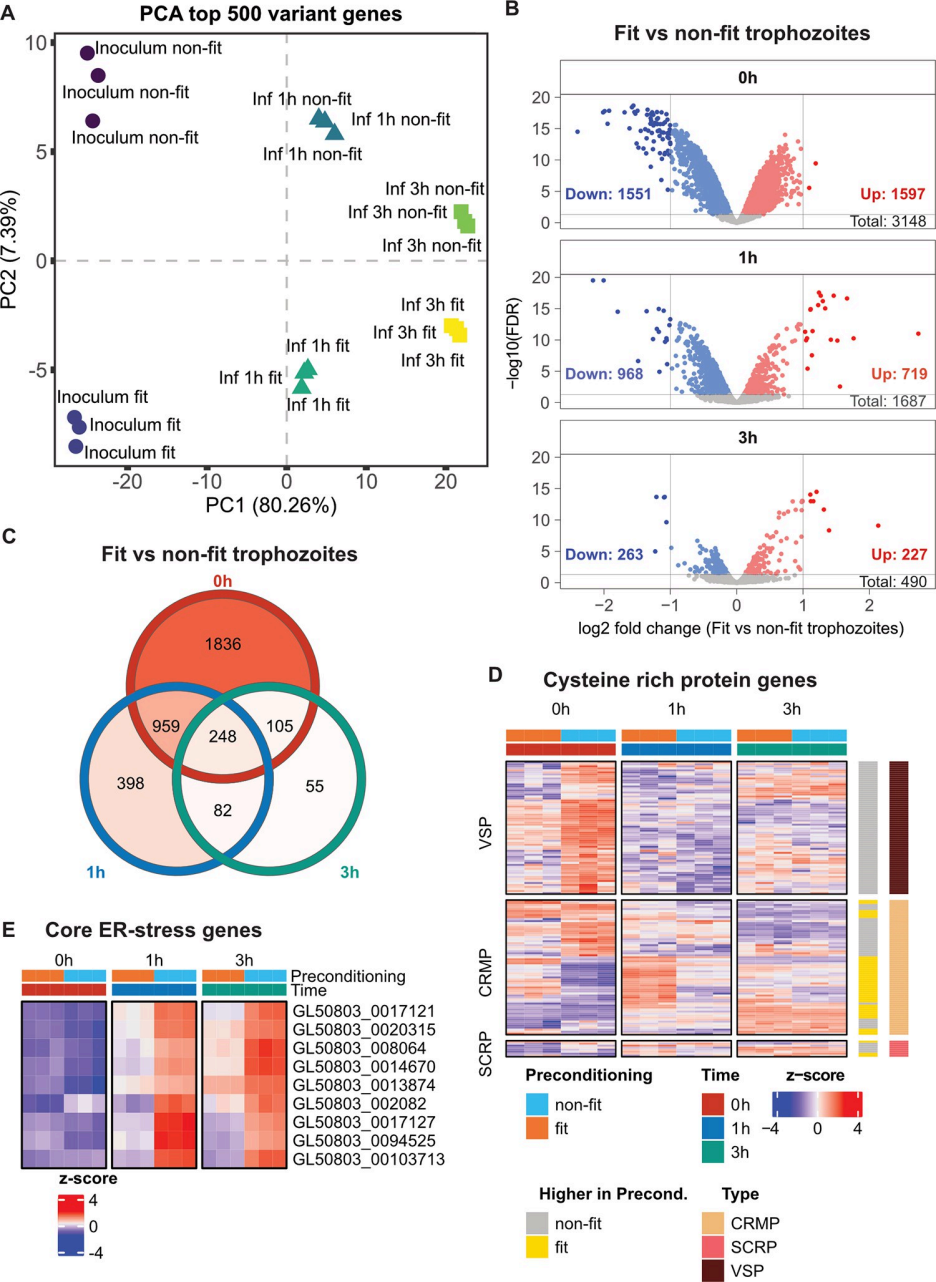
Transcriptome analysis of *Giardia intestinalis* trophozoites after preconditioning and during intestinal epithelial cell infection. (A) Plot of the principle component analysis (PCA) illustrating PC1 and PC2 of the top 500 *G*. *intestinalis* variant genes. (n = 3 biological replicates). (B) Volcano plots showing differentially expressed *G*. *intestinalis* genes between “fit” (DMEM/F-12+FBS) and “non-fit” (DMEM/F-12) samples at 0h (inoculum), 1h and 3h post infection (p.i.) (n = 3 biological replicates). Each dot represents a differentially expressed gene (DEG) and are coloured according to expression fold change and false discovery rate (FDR) (grey, FDR > 0.05; dark blue FDR < 0.05 and log2FC < -1; light blue FDR < 0.05 and log2FC < 0; dark red FDR < 0.05 and log2FC > 1, light red FDR < 0.05 and log2FC > 0). (C) Venn diagram of differentially expressed *G*. *intestinalis* genes between “fit” (DMEM/F-12+FBS) and “non-fit” (DMEM/F-12) samples at 0 h, 1 h and 3 h p.i.. (D) Heatmap showing the expression of cysteine rich protein genes that are differentially expressed between “fit” and “non-fit” inoculum samples at 0 h. Genes annotated in grey are higher expressed in “non-fit” trophozoites and genes annotated in yellow are higher expressed in “fit” trophozoites. Expression values are z-score transformed transcript counts. (E) Heatmap showing core ER-stress genes. Expression values are z-score transformed transcript counts. VSP, variant-specific surface protein; CRMP, cysteine rich membrane protein; SCRP, secreted cysteine rich protein.

Finally, we analysed *G*. *intestinalis* gene expression changes after preconditioning (DMEM/F-12+FBS vs DMEM/F-12) and during the progression of the IEC monolayer infection. Principle component analysis of the top 500 *G*. *intestinalis* variant genes showed a clear separation of sample groups by time (PC1) and by type of preconditioning (PC2) (Fig 5A). PC2 variance, explaining *G*. *intestinalis* trophozoite preconditioning, became progressively smaller over the infection period, showing that transcriptional programs in “fit” and “non-fit” trophozoites gradually became more similar atop the enteroid-derived IEC monolayers (Fig 5A). This was also reflected in the extent of differential gene expression, with 1687 and 490 DEGs noted between “fit” and “non-fit” *G*. *intestinalis* after 1h and 3h p.i., respectively, compared to a striking 3148 DEGs prior to the infection (Fig 5B and 5C, S4 Table). The expression levels of individual genes differentially expressed between “fit” and “non-fit” trophozoites also became more similar over the course of the infection (S9C Fig). In accordance with the change of the trophozoites’ expression profile during the infection, differential gene expression analysis of trophozoites during the infection compared to their respective infection inoculum showed strong transcriptional changes both after 1 and 3h p.i. (>> 3400 DEGs in any infection and inoculum comparison) (S9A Fig, S4 Table). The majority of these DEGs, 2382 genes, overlapped between the infection time points and condition (S9B Fig).

Interestingly, VSPs, immunodominant surface proteins involved in antigenic variation [53–55], comprised DEGs selectively upregulated in the “non-fit” trophozoite inoculum (Figs 5D and S9D). Other cysteine rich proteins, cysteine rich membrane proteins (CRMP) and secreted cysteine rich proteins (SCRP), were also differentially expressed between “fit” and “non-fit” trophozoites, but with less obvious overall trends (Fig 5D). Of further note, during the infection, there was a pronounced difference in expression of ER-stress related genes between “fit” and “non-fit” trophozoites (Fig 5E). All nine core ER-stress genes [56] were expressed to a markedly higher level by “non-fit” trophozoites, at both 1h and 3h p.i. (Fig 5E). Taken together, these data suggest that “non-fit” *G*. *intestinalis* trophozoites feature dramatically altered transcription of surface protein genes, combined with persistent secretory pathway stress upon encounter of the epithelium.

## Discussion

In this study, we validated that human enteroid-derived IEC monolayers are a suitable model to study host *G*. *intestinalis* interactions, particularly the early host cell immune responses against the parasite. We showed that in this infection model system, *G*. *intestinalis* trophozoites attach stably to the IEC surface, establish an infection and trigger a variable IEC immune response. Most notably, we found that the trophozoite fitness dramatically changes the parasite’s interaction with host IECs and therefore likely also the outcome of the infection. Specifically, we could observe that “non-fit” trophozoites, which were preincubated in serum-deprived media for 1 h before the infection, caused a pronounced cytokine transcript response in IEC monolayers, compared to “fit” trophozoites that were preincubated in media supplemented with serum (Fig 2C and 2D). The IEC response elicited by “non-fit” *G*. *intestinalis* was not scalable, instead infections with a higher MOI resulted in lower cytokine mRNA upregulation in IECs, resembling more the “fit” trophozoite phenotype (Fig 3A). We further showed that “fit” trophozoites can actively down-regulate IEC cytokine transcript responses to highly immunostimulatory trophozoite lysates. This finding of an immunosuppressive capacity of the parasite is consistent with human infection data where *G*. *intestinalis* has been observed to cause little or no mucosal inflammation [17]. The low host inflammatory response does in most cases not correlate with disease pathogenesis and diarrhoea [57]. *G*. *intestinalis* can cause acute and asymptomatic, but also chronic infections, even in immunocompetent patients [11]. Recent studies have also indicated that *G*. *intestinalis* can actively suppress or modulate host inflammatory responses under other conditions. Bone marrow-derived dendritic cells (BMDCs) incubated with *G*. *intestinalis* extracts decreased expression of IL12 and increased expression of anti-inflammatory IL10 when stimulated with TLR ligands [58]. Further, nitric oxide (NO) production is impaired in macrophages [59] and IECs [60] during *G*. *intestinalis in vitro* infections. Cotton *et al*. showed that assemblage A *G*. *intestinalis* infection in mice caused decreased granulocyte infiltration into colonic tissue and decreased cytokine expression after intra-rectal instillation of *Clostridium difficile* toxin A and B [18]. Moreover, a recent study showed that *Giardia muris* can reduce enteropathogen (*Citrobacter rodentium)* induced colitis by increasing antimicrobial peptide production in mice [19]. Dann *et al*. also demonstrated that the parasite can regulate inflammation; IL10 knockout mice infected with *Giardia muris* exhibited increased colonic inflammation, while there was no change at the site of infection in the small intestine [61]. Similar observations have been made in human patients where inflammation was seen in the duodenum in the absence of parasites, whereas the heavily parasite-infested ileum did not show signs of inflammation [62]. In contrast to these findings of immune suppression, enteric co-infection with *G*. *intestinalis*, assemblage B, and enteroaggregative *Escherichia coli* (EAEC) caused exacerbated malnutrition in mice on a low-protein diet, and preservation of the inflammatory response to EAEC [63]. The immunomodulatory capacity of the parasite might be both *G*. *intestinalis* strain and host specific, i.e., based on the nutritional status, immune competency, and microbiome composition. Importantly, our study demonstrates for the first time that “fit” *G*. *intestinalis* trophozoites can directly suppress cytokine transcriptional expression in non-transformed human small intestinal IECs. As these cells are important for early innate immune responses and typically constitute the first cell type that the parasite encounters after excystation, we need to better understand how *G*. *intestinalis* trophozoites are recognized and elicit/suppress immune responses, such as cytokine and chemokine expression, at this barrier. This includes also teasing apart the multi-layered effects of the parasite on not only IEC transcript responses, but also on extracellular cytokine protein levels over time (Figs 1, 2 and S4).

RNA sequencing of *G*. *intestinalis* infected IECs again revealed a stronger host response to “non-fit” rather than to “fit” parasites, already after 1 h, and even more so after 3 h of infection (Fig 4A and 4B). Despite the marked difference in the number of DEGs, we could observe that both parasite phenotypes caused a pro-inflammatory cytokine upregulation. However, “fit” trophozoites elicited a lower upregulation of all identified cytokines in this class (S1 Table). We further found that immune regulating genes, e.g. *SOCS1-3*, *ZC3H12A*, *BCL3* and *TNFAIP3* were upregulated during infections with both “fit” and “non-fit” trophozoites, while “non-fit” trophozoites also upregulated the immune-regulatory genes encoding *TNFAIP6*, *MIR221*, *NFKBID*, *ZFP36L1*, *IL10RA* and *ZBP1* (Fig 4C, S2 Table). An increased expression of the necrosis regulator and innate immune sensor, ZBP1, has previously been correlated with the activation of RIPK1/RIPK3 and NF-κB [64], as well as an increased IFN-α/β expression [65]. This is the first observation that *ZBP1* expression is upregulated during *G*. *intestinalis* infection and it will be of significant interest to investigate if *ZBP1* is part of the host immune signalling response in this context. Taken together, our IEC transcriptome analysis demonstrates that both the total number of DEGs, and their respective expression magnitude, is dictated by the fitness of the infecting *G*. *intestinalis* trophozoites.

The parallel parasite RNA sequencing revealed extensive transcriptional changes between differentially preconditioned trophozoites, dependent on if they were preincubated with or without FBS. “Non-fit” trophozoites (-FBS) seemed to exhibit membrane stress (S3F Fig), ER-stress (Fig 5E), as well as upregulation of many VSPs (Fig 5D), indicating potential VSP switching event(s) in a sup-population of trophozoites. The serum-deprivation causes a lack of lipids in the culture media. Earlier work have shown that lipids and fatty acids influence the parasite’s growth and differentiation [48,49,66]. Media supplementation with biliary lipids has also been shown to support trophozoite growth in serum-deprived media [49]. *G*. *intestinalis* takes up most essential lipids from the environment and can only synthesise certain phospholipids by itself [67,68]. Recent studies have shown altered lipid parameters in patients infected with *G*. *intestinalis* [69,70]. Of further interest, a high fat diet in mice increases the severity of *G*. *intestinalis* assemblage B infection and the trophozoite burden in the small intestine. Mice on a high fat diet also had more mucosal infiltration of inflammatory cells following *G*. *intestinalis* infections [71]. Cholesterol availability is important for lipid raft assembly in *G*. *intestinalis* and lipid rafts have recently been linked to virulence and pathogenicity [72]. In culture, cholesterol-binding compounds blocked giardial lipid raft formation [73], and treatment with nystatin, a cholesterol-binding agent, inhibited encystation *in vitro* [73], and changed the protein distribution patterns, there among many virulence factors, in the parasites’ extracellular vesicles (EVs) [72]. In our infection model, the starvation of cholesterol during trophozoite preconditioning might therefore cause a disruption of lipid raft formation and/or change the protein distribution in EVs produced by “non-fit” vs “fit” trophozoites. The altered transcription of surface protein genes, encoding e.g. VSPs, might further explain why “non-fit” trophozoites elicit a potent transcriptional immune response in human IECs, while their “fit” counterparts can evade, and even suppress, this response.

RNA-seq further revealed that the transcriptional expression of “non-fit” and “fit” *G*. *intestinalis* trophozoites became more similar over the course of the early infection. As trophozoites are incubated in the same serum-deprived media during the infection, it appears likely that they obtain their essential lipids from the host during active infection. GO term analysis showed that cholesterol biosynthetic processes in the host IECs were indeed downregulated 3 h after infection with “non-fit” trophozoites (Fig 4D), indicating a disturbed cholesterol homeostasis. Future studies should aim to assess lipid metabolism and homeostasis during *G*. *intestinalis* infection and what effect this has on the outcome of the infection.

Recently, Holthaus *et al*. developed an enteroid-based transwell infection system to study *G*. *intestinalis* IEC interactions and characterize intestinal barrier dysfunction [32]. The three biggest differences between our study and the Holthaus *et al*. study are that they i) used enteroids derived from the duodenum, ii) *Giardia* growth media (TYDK) as the apical infection medium, and iii) did not precondition trophozoites before infections. TYDK is supplemented with 10% bovine serum and is a nutrient-rich, bile-containing and reducing culture medium, containing among other things cysteine, ferric ammonium citrate, ascorbic acid, yeast extract, peptone and bovine bile. In such a rich culture media, which is optimised for *G*. *intestinalis* axenic *in vitro* growth, the parasite has access to all essential nutrients and supplements. Thus, the trophozoites will most likely not be deficient for either micronutrients or lipids and should therefore be maximally “fit”. The authors could indeed not observe any IEC transcriptional changes during the first 1.5 h of infection, but they could see a response after 24 h of infection. After 24 h much of the nutrients, such as arginine, will be depleted by the parasite [23]. This supports our main conclusion that trophozoite fitness and the nutrient availability is crucial for the outcome of *G*. *intestinalis* infection. This further highlights that it is important to choose the infection model system carefully when studying the early host immune responses against *G*. *intestinalis* trophozoites. The infection method we describe here will therefore aid in future immune response studies using human enteroids and other organoid-based models, and where the early stage of the infection is the primary focus. However, there is room for further improvement in the complexity of this experimental *G*. *intestinalis* infection model system, e.g. by implementing organoid-immune cell co-cultures, as has been done for other pathogens [74–77]. Moreover, the newly introduced *G*. *intestinalis* adapted CRISPR/Cas9 method will make it possible to efficiently create gene knockouts in the parasite [78], as well as in the IECs [79]. The combination of genetically modified *G*. *intestinalis* and enteroid/organoid infection model systems should greatly aid in the identification of more specific host-parasite molecular interactions, and lead to the definitive unravelling of the parasite’s elusive immunomodulating mechanisms.

## Materials and methods

### Ethics statement

Human jejunal enteroid cultures from two independent donors were established from resected tissues acquired from routine bariatric surgery. Subjects gave their informed written consent. All personal information was pseudonymized before reaching the laboratory and researchers did not know the patients’ identities. The procedures were approved by the local governing body, Etikprövningsmyndigheten, Uppsala, Sweden, under the license number 2010–157 with addenda 2010-157-1 (2018-06-13) and 2020–05754 (2020-10-26).

### Enteroid culture

The enteroid cultures (pseudonym IDs 18–8 and 18–9) used in this study were established and maintained as described before [33,42,80]. Briefly, resected human jejunal tissue was washed in ice-cold phosphate-buffered saline (PBS), followed by dissociation of epithelial crypts using gentle cell dissociation reagent (STEMCELL Technologies, Vancouver, BC, Canada). Next, epithelial fragments were filtered through a 70-μm cell strainer and crypt-enriched fractions were embedded in 50 μl Matrigel (Corning, Corning, NY, USA) domes, which were cultured after solidification in OGM (IntestiCult organoid growth medium [Human], STEMCELL Technologies) with 100 U/ml penicillin-streptomycin (Thermo Fisher [Gibco], Waltham, MA, USA) at 37°C and 5% CO_2_. During the first 1–2 days of culture 10 μM Y-27632 (Sigma Aldrich or STEMCELL Technologies) was added to the embedded crypts and the growth medium was refreshed every 2 to 3 days. The human enteroids were maintained by passaging weekly at a ratio of ~ 1:8 by mechanical dissociation. The Matrigel domes were disrupted by pipetting with gentle cell dissociation reagent and then washed once with Dulbecco’s modified Eagle’s medium (DMEM)/F-12 (cat# 11330057, Thermo Fisher [Gibco])–1.25% bovine serum albumin (BSA) (Thermo Fisher [Gibco]). The suspended enteroids were further disrupted by pipetting with a 200-μl pipette tip. The enteroid fragments were resuspended in 50μl Matrigel/OGM, at a ratio of 3:1, and divided over 3 domes per well in a 24-well plate. The cultures were kept at 37°C and 5% CO_2_ and experiments were conducted at enteroid passage number 3–30.

### Enteroid microinjection

*Giardia intestinalis* microinjections into human enteroids were performed in expanding enteroids at 4–5 days post-seeding. Enteroids were passaged as described above and embedded in 50 μl elongated, loaf-shaped 90–100% Matrigel domes seeded in a 35-mm glass-bottom dish (no. 1.5 coverslip, 20-mm glass diameter, uncoated, MatTek P35G-1.5-20-C). Non-optimised (bacteria-adapted) microinjection needles were prepared as described before [42]. Briefly, 1.0-mm filamented glass capillaries (World Precision Instruments, no. BF100-78-10, Borosilicate, 1 mm wide, 100 mm long, with filament) were used in a micropipette puller (Sutter Instruments, P-1000; settings: heat = ramp + 5, pull  =  60, velocity  =  80, delay  =  110, pressure  =  200). The pulled needles were afterwards bevelled at a 30° angle on a fine-grit diamond lapping wheel. The *G*. *intestinalis*-optimised microinjection needles were also prepared using 1.0-mm filamented glass capillaries (World Precision Instruments, no. BF100-78-10, Borosilicate, 1 mm wide, 100 mm long, with filament) and a micropipette puller using modified settings (Sutter Instruments, P-1000; settings: heat = ramp + 15, pull  =  90, velocity  =  70, delay  =  90, pressure  =  200). Next, the needle tip was cut ~0.5mm from the end using a razor blade followed by bevelling at a 30° angle on a fine-grit diamond lapping wheel. The *G*. *intestinalis* trophozoite inoculum (~1x10^8^ cells/ml) was loaded into the microinjection needles by fluidic force. Microinjections were performed using a microinjector (MINJ-FLY, Tritech Research) and a micromanipulator (uMP-4, Senapex) employing a 0.02 to 0.2-s air pressure pulse.

### Enteroid-derived IEC monolayer culture

The enteroid-derived monolayers were cultured on 24-well transparent polyethylene terephthalate (PET) tissue culture inserts with 0.4-mm pores (Sarstedt, Nümbrecht, Germany) or for DIC live imaging on 13-mm-diameter alumina Whatman Anodisc membranes with 0.2-μm pores (GE Healthcare, Little Chalfont, United Kingdom) in so called apical imaging chambers (AICs) [33]. The coating and preparation of transwells were done as described before [33]. Briefly, PET transwell inserts were coated with 40x diluted Matrigel in PBS for 1h at room temperature before seeding. The coating solution was completely removed and the cell suspension was immediately added to the transwell inserts. Alumina membranes were incubated in 30% H_2_O_2_ for 1h at room temperature, followed by washing in sterile distilled water (dH_2_O) and incubation in 0.1 mg/ml poly-L-lysine (Sigma-Aldrich, Stockholm, Sweden) in dH_2_O for 5 min. The alumina membranes were air-dried and coated with 40x diluted Matrigel in dH_2_O for 1 h and air-dried again. After coating, the membranes were mounted within AICs as described before [33].

Human enteroids were dissociated into single cells as described previously [80]. Briefly, enteroids were dissociated 7 days after passaging. The Matrigel embedded enteroids were first broken up in gentle dissociation reagent and then washed once in DMEM/F-12/1.25% BSA. Enteroids were dissociated into single cells using TrypLE Express (Thermo Fisher [Gibco]) for 5–10 min at 37°C followed by extensive pipetting. Single cells were pelleted by centrifugation at 300 x g for 5 min and resuspended in OGM+Y (Rho kinase inhibitor Y-27632 [10 μM]). The cells were counted and diluted to seed 3.0 x 10^5^ cells into the apical compartment of PET transwells in 150 μl (600 μl medium in the basal compartment, 24-well plate wells) or into the apical compartment of AICs in 75 μl (600 μl medium in the basal compartment, 12-well plate wells). The Y-27632 inhibitor was removed from the culture media after 2–3 days. The monolayers reached confluency in ~2 to 4 days after seeding. Confluent monolayers were then differentiated towards an enterocyte phenotype by deprivation of WNT signaling for 4 to 5 days. The differentiation medium (ENR) consisted of DMEM/F-12 supplemented with 5% R-Spondin1 conditioned medium (home made from Cultrex 293T R-spondin1-expressing cells; R&D Systems, MN, USA), 10% Noggin conditioned medium (home made with HEK293-mNoggin-Fc cells; kindly provided by Hans Clevers, Utrecht University), 50 ng/ml mouse recombinant EGF (Sigma-Aldrich), 1x B27 supplement (Thermo Fisher [Gibco]), 1.25 mM N-acetyl cysteine, and 100 U/ml penicillin-streptomycin [34,37].

Transepithelial electric resistance (TEER) measurements were conducted during enteroid-derived IEC monolayer growth using an EVOM2 Epithelial Voltohmmeter (World Precision Instruments) equipped with a STX2 electrode (World Precision Instruments). Blank electric resistance (cell-free transwell insert containing only culture media) was subtracted from raw resistance values followed by standardization for 1 cm^2^ surface area.

### *Giardia intestinalis* culture, preconditioning and infection

All *Giardia* infections in this study were performed with *Giardia intestinalis* isolate WB, clone C6 (ATCC 50803). For fluorescence microscopy experiments a *G*. *intestinalis* line constitutively expressing mNeonGreen was used [33]. *G*. *intestinalis* trophozoites were grown at 37°C in 10-ml flat plastic tubes (Thermo Fisher [Nunc], MA, USA) or in 15- or 50-ml tubes (Sarstedt) filled with TYDK medium (also known as modified TYI-S-33 or Keister’s medium) [81], supplemented with 10% heat-inactivated bovine serum (Thermo Fisher [Gibco]). Materials used in the TYDK medium were purchased from Sigma-Aldrich (MO, USA). *G*. *intestinalis* trophozoites were grown to ~ 70% confluence for IEC monolayer infections. Before the infection, trophozoites were washed twice with warm 1xPBS to remove dead cells and then incubated in preconditioning media, DMEM (cat# D6546, Sigma-Aldrich) supplemented with 10% heat-inactivated FBS (Thermo Fisher [Gibco]) (DMEM+FBS), for 1 h or 2 h, or in DMEM, DMEM/F-12, DMEM/F-12 supplemented with 10% Heat-Inactivated FBS (DMEM/F-12+FBS) or TYDK for 1h. Next, trophozoites were incubated on ice for 12 min, counted and pelleted by centrifugation (800 x g, 10 min, 4°C). The pellets of all preconditioning conditions were washed once in 1 ml DMEM/F-12 to remove any preconditioning media, centrifuged and diluted again in DMEM/F-12. Therefore, all infections were performed in the same infection medium (DMEM/F-12) and no supernatants of preconditioned trophozoites were included. PET-transwell IEC monolayer, ENR differentiated, infections were performed using 3.96 x 10^5^ trophozoites (MOI1.2) or 3.3 x 10^6^ (MOI10) trophozoites with an inoculum volume of 50 μl. PET-transwell IEC monolayer, OGM undifferentiated, infections were performed using 7.92 x 10^5^ trophozoites (MOI1.6) trophozoites with an inoculum volume of 50 μl. Heat-inactivated and lysed trophozoites were prepared as described above using the preconditioning media used in the respective experiment (TYDK in Fig 2E, DMEM/F-12+FBS in Figs 2F, 3B and S4). Trophozoites were diluted in DMEM/F-12 according to an inoculum of MOI1.2 (3.96 x 10^5^ trophozoites). Next, the cells were incubated at 60°C for 30 min for heat-inactivation or sonicated (2 min 100% amplitude, 30 pulse, 10 sec break) for lysed trophozoites. PET-transwell-grown IEC monolayers were inoculated with 50μl *G*. *intestinalis* lysate or heat-inactivated slurry. Trophozoite attachment to IECs during enteroid monolayer infections were determined by counting trophozoites (Bürker chamber) in the apical supernatant of the monolayers at the given time point of infection and comparing to the inoculum before the infection. For alumina membrane IEC monolayer infections (ENR differentiated), trophozoites were preincubated with DMEM+FBS for 30 min and all other preparation steps were followed as described above. Infections were done at MOI1.2 (3.96 x 10^5^ trophozoites) with an inoculum volume of 10 μl.

For microinjections *G*. *intestinalis* trophozoites were grown in TYDK in 50-ml tubes as described above to a confluency of 70%. Before the microinjection, the trophozoites’ growth media (TYDK) was decanted and replaced with fresh TYDK to remove dead cells. Cells were detached on ice for 12 min, counted and pelleted by centrifugation (800 x g, 10 min, 4°C). Trophozoites were resuspended to ~1x10^8^ cells/ml in TYDK.

### *Salmonella* Typhimurium culture and infection

*Salmonella* enterica serovar Typhimurium, SL1344 (SB300), was used in this study [82]. For IEC monolayer infections, *Salmonella* was grown in LB/0.3 M NaCl (Sigma-Aldrich) for 12 h overnight, supplemented with Streptomycin (100 μg/ml). The following day, a 1:20 dilution was subcultured in LB/0.3 M NaCl without antibiotics for 4 h. For IEC monolayer infections, the 4 h sub-culture was diluted in DMEM/F-12 without antibiotics for a final MOI of 10 (50 μl inoculum volume).

### Live-cell infection imaging and image processing

Imaging of AIC monolayers was performed as described before [33] on a custom built upright microscope. The upright microscope is based on the Thorlabs Cerna upright microscopy system (Newton, NJ, USA), with a heated 60x/1.0 NA Nikon CFI APO NIR objective (2.8 mm WD) and a Nikon d-CUO DIC oil condenser (1.4 NA), controlled by Micro-Manager 2.0-gamma [83]. Images were obtained using an ORCA-Fusion camera (model number C14440-20UP; Hamamatsu Photonics, Hamamatsu City, Japan), with a final pixel size of 109 nm. Transmitted light was supplied by a 530-nm Thorlabs LED (M530L3) to limit phototoxicity and chromatic aberrations. The microscope chamber was kept during the infection at 37°C in a moisturized 5% CO_2_ atmosphere. IEC monolayers in AICs were placed in 35-mm glass-bottom dishes (Cellvis, Mountain View, CA, USA) in pre-warmed 3 ml DMEM/F-12 without antibiotics in the microscope’s light path 30 min before the infection. *G*. *intestinalis* was added in premade dilutions directly underneath the objective, and the imaging was started immediately. PET-transwell IEC monolayers and enteroid microinjections were imaged using an inverted custom-built microscope. The inverted microscope is based on an Eclipse Ti2 body (Nikon), using 10x, 40x or 60× (0.45, 0.6, 0.7 numerical aperture, respectively) Plan Apo Lambda air objectives (Nikon) and a back-lit sCMOS (scientific complementary metal oxide semiconductor) camera with a pixel size of 11 μm (Prime 95B; Teledyne Photometrics). Fluorescence images were obtained using the excitation light engine Spectra-X (Lumencor) and emission collection through a quadruple band pass filter (89402; Chroma). Imaging was performed in a microscope chamber at 37°C in a moisturized 5% CO_2_ atmosphere.

The obtained microscopy images were processed using Fiji [84]. DIC images were filtered to acquire an even field of illumination by subtracting a (30-pixel sigma) Gaussian blurred projection from the original as described before [33].

### RNA extraction, library preparation, and RNA sequencing

All samples were collected using TRIzol Reagent (Thermo Fisher) with 300 μl reagent for transwell samples and 1 ml reagent for axenic trophozoite cultures. RNA extractions were performed according to the manufactures protocol. Purified RNA was DNaseI (Thermo Fisher) treated to remove genomic DNA. 500 ng total RNA per sample was used for the sequencing library, employing the TruSeq stranded mRNA library preparation kit (cat# 20020595, Illumina Inc., CA, USA) including polyA selection. Unique dual indexes (cat# 20022371, Illumina Inc.) were used for the library. The library preparation was performed according to the manufacturers’ protocol (#1000000040498). The quality of the libraries was assessed using a TapeStation (Agilent Technologies, CA, USA) and the D1000 Screen Tape. The adapter-ligated fragments were quantified by qPCR using the library quantification kit for Illumina (KAPA Biosystems, UK) and a CFX384 Touch instrument (Bio-Rad Laboratories, CA, USA) prior to cluster generation and sequencing. Sequencing was performed on an Illumina NovaSeq 6000 instrument (NSCS v 1.7.0/ RTA v 3.4.4) according to the manufacturer’s instructions. Demultiplexing and conversion to FASTQ format was done using the bcl2fastq2 (2.20.0.422) software, provided by Illumina. Additional statistics on sequencing quality were compiled with an in-house script from the FASTQ-files, RTA and BCL2FASTQ2 output files. Sequencing was performed by the SNP&SEQ Technology Platform, Science for Life Laboratory (SciLifeLab), Uppsala, Sweden.

### Bioinformatics analyses of RNA sequencing data

Our own dual RNA sequencing (RNA-seq) pipeline was used for analysing differentially expressed genes for both *G*. *intestinalis* and human IECs, as described before [22]. Scripts of the bioinformatics analysis used in this study are available upon request. Briefly, STAR v2.7.10a [85] was used to map the RNA-seq read counts to the *G*. *intestinalis* WBC6 reference genome (GCA_000002435.2UU_WB_2.1) [86] and to the human genome (GRCh38.p12, GCA_000001405.28). The STAR parameter “–quantMode GeneCounts” was used to obtain the raw counts per gene which were used for downstream analysis. All further data analysis was done in R (v 4.0.1). Differential gene expression analysis was performed with the edgeR (v 3.32.1) [87] package with the quasi-likelihood (QL) F-test (glmQLFTest) to determine significant differential gene expression. Genes were considered significant when the false discovery rate (FDR) ≤ 0.05. Reactome pathway, GO term (Molecular Function, Biological Process) enrichment analysis and gene set enrichment analysis were done on significant DEGs using clusterProfiler (v3.18.1) [88]. Venn diagrams were constructed with the ggVennDiagram package (v 1.2.0) [89] and heatmaps were produced with the ComplexHeatmap package (v2.6.2) [90]. VSP gene counts were normalized using gene length-corrected trimmed mean of M-values (GeTMM) [91] in S9D Fig. Raw reads and processed raw counts per gene were deposited at Gene Expression Omnibus (GEO), available as accession ID GSE220954. Data from the RNA seq analyses can also be found in S1–S4 Tables.

### Quantitative Polymerase Chain Reaction (qPCR)

High-quality and DNaseI treated RNA samples were reverse transcribed to cDNA using the Revert Aid H Minus cDNA Synthesis Kit (Thermo Fisher) with oligo-(dT)18 primers, according to the manufacturer’s instructions. qPCR was performed using 2 ng cDNA template per reaction, 0.3μM of each primer and the SsoAdvanced Universal SYBR Green Supermix (2x) (Bio-Rad Laboratories). Reactions were set up in 10 μl volumes and run on a Bio-Rad CFX 384 instrument according to the manufacturer’s protocol. qPCR primers used in this study are listed in S5 Table. For all qPCRs, GAPDH was used as the housekeeping gene. The fold change values in gene expression were calculated using the 2^−ΔΔCT^ method [92]. Statistical significance in RNA levels was determined using Welchs’ *t*-test with Holm-type corrections for multiple testing.

### Enzyme-linked immunosorbent assay (ELISA)

ELISAs were performed to measure cytokine release from the IEC monolayers in both the apical and basal compartment supernatants. IEC monolayers were infected with *G*. *intestinalis* trophozoites preconditioned with DMEM/F-12 or DMEM/F-12+FBS, or with trophozoite cell lysis as described above for 3 h and 6 h at MOI1.2. The ELISAs were performed on spent media from infection samples and uninfected control samples at the respective time points. The medium was collected, centrifuged (1000 x g, 10 min, 4°C) to remove cellular debris and stored at -20°C until analysis. The assayed cytokines included CXCL1 and CXCL8. CXCL1 was measured using the Human CXCL1/GRO alpha Quantikine ELISA Kit (R&D Systems) and CXCL8 was measured using the IL-8 Human ELISA Kit (cat# KHC0081, Thermo Fisher [Invitrogen]) according to the manufacturer’s instructions. Absorbance reads from samples and standard curves were plotted in GraphPad Prism version 9.2.0, GraphPad Software, San Diego, California USA, www. graphpad.com. Cytokine concentrations were obtained by using a four-parameter logistic ELISA curve fitting. Statistical significance in protein concentrations was determined using a one-way analysis of variance (ANOVA) at α < 0.05, followed by Bonferroni comparisons (*P* < 0.05).

### Trophozoites’ ATP concentration, propidium iodide staining, and axenic growth

*G*. *intestinalis* trophozoites’ intracellular ATP levels were measured using CellTiter-Glo Luminescent Cell Viability Assay (Promega, WI, USA) according to the manufacturer’s protocol. Briefly, trophozoites were grown in TYDK to ~70% confluence, followed by preconditioning in TYDK, DMEM/F-12+FBS, or DMEM/F-12 for 1h, as described above. After the preconditioning the trophozoites were detached by incubation on ice for 12 min, then the trophozoites were counted, pelleted and resuspended at 1x10^6^ trophozoites/ml in preconditioning media. 200 μl/per well of trophozoite suspension was seeded in a 96-well plate and incubated for 15 min at room temperature. Media only negative controls were included for all preconditioning media. Next, 50 μl CellTiter-Glo reagent was added, the solutions were mixed by shaking for 10 min. The solutions were then allowed to settle for 10 min after which the luminescence was read using a Tecan plate reader (Infinite M200 Pro).

The integrity of the trophozoites´ cell membrane was assessed using propidium iodide (PI) staining. Trophozoites were grown and preconditioned as described above (TYDK, DMEM/F-12+FBS or DMEM/F-12), followed by detachment (12 min in ice), pelleting (centrifugation: 800 x g, 10 min, 4°C) and resuspension in 1.5 ml DMEM/F-12. Trophozoites were then stained with 40 μl propidium iodide Ready Flow Reagent (Thermo Fisher [Invitrogen]) for 15 min at room temperature. Positive controls were prepared as described above, but the trophozoite pellets were taken up in DMEM/F-12 supplemented with 0.1% Triton x-100 (Sigma-Aldrich). Fluorescence of the trophozoites were assessed using a MACSQuant VYB flow cytometer (Miltenyi Biotec, Germany) using the yellow laser (561 nm) and Y2 filter (615 nm/20 nm).

Growth differences during the 1h preconditioning in TYDK, DMEM/F-12+FBS or DMEM/F-12 was determined by cell counting. Briefly, trophozoites were grown in TYDK to ~50% confluency, followed by preconditioning as described above for 1h. Next, trophozoites were counted using a Bürker chamber.

## Supporting information

S1 Fig 3D enteroid infection by microinjection of *Giardia intestinalis* trophozoites expressing mNeonGreen. (A) *G*. *intestinalis* microinjection with a non-optimised microinjection needle (<7μm tip diameter) caused trophozoites to rupture and clog the needle. Scale bar = 50 μm. (B) Tips of optimised *G*. *intestinalis* microinjection needles with wider openings (7–9 μm diameter). Scale bar = 10 μm. (C) Microinjection of a 3D enteroid with an optimised *G*. *intestinalis* needle that is clogged by *G*. *intestinalis* trophozoites. Scale bar = 50 μm. (D) Representative 3D enteroid successfully microinjected with *G*. *intestinalis*-mNeonGreen trophozoites imaged over the course of the infection until the enteroid collapsed. Time post infection is indicated as hours: minutes: seconds. Scale bar = 50 μm. (E) Microinjection of *G*. *intestinalis*-mNeonGreen trophozoites into 3D enteroids visualized by DIC and fluorescence microscopy imaging (upper and lower panel, respectively). Time post infection is indicated as hours: minutes: seconds. (F) Time-lapse microscopy of trophozoites in the mNeonGreen fluorescence channel. Manual tracking of a single trophozoite swimming atop the IEC surface inside the enteroid. The tracked trophozoite is indicated by magenta circles and the track by yellow lines. Representative attached trophozoite is indicated by a red cross. Scale bar = 10 μm.
(TIF)

S2 Fig Characterization of IEC monolayers and validation of DMEM/F-12 as an infection medium in both apical and basal compartments. (A) Human jejunal enteroid IEC monolayers were grown on PET-transwells and TEER measurements were performed to verify an increase in confluency and barrier integrity over time (n = 4 biological replicates). The black dotted line indicates the medium change to differentiation media (ENR) at day 5 after seeding and the red dotted line indicates the day of infection at day 10 after seeding. (B) Schematic representation showing the jejunal enteroid-derived IEC monolayer infection model, using only DMEM/F-12 as the infection media. Created with BioRender.com. (C) *G*. *intestinalis* trophozoite attachment to IECs over the course of the monolayer infection. (D) qPCR of host cell response genes at 30 min, 1.5 h, 3 h and 4.5 h post *G*. *intestinalis* infection, including 0h and 4.5h negative controls (n = 3 biological replicates ± SD). Fold change values of all samples were calculated by comparing to the 0 h uninfected control. *G*.*i*., *Giardia intestinalis*; ENR, enterocyte differentiation media; TYDK, *G*. *intestinalis* growth media.
(TIF)

S3 Fig Media preconditioning of *Giardia intestinalis* trophozoites alter their phenotype. (A) *G*. *intestinalis* trophozoite attachment to IECs after 1h of infection. Trophozoites were preconditioned with DMEM+FBS, DMEM, DMEM/F-12+FBS, DMEM/F-12, or TYDK for 1h before the infection. (B) Preconditioned trophozoite (DMEM/F-12+FBS or DMEM/F-12) infections (MOI1.2) of enteroid-derived IEC monolayers established from another human donor culture. qPCR of chemokine mRNA expression levels at 1 h post *G*. *intestinalis* infection (n = 4 biological replicates, except n = 5 for 1 h *G*. *intestinalis* DMEM/F-12 preconditioning infection ± SD). Fold change values of all samples were calculated by comparing to the 1 h uninfected control. (C) qPCR of chemokine mRNA expression levels in IECs infected for 1 h with *G*. *intestinalis* trophozoites preconditioned with DMEM+FBS either for 1 h, or 2 h (n = 3 biological replicates ± SD). Fold change values of all samples were calculated by comparing to the 1 h uninfected control. (D) Trophozoite growth after 1 h of preconditioning in DMEM/F-12, DMEM/F-12+FBS or TYDK (n = 3 biological replicates). (E) Intracellular ATP levels of trophozoites after 1h of preconditioning measured with CellTiter-Glo cell viability assay (n = 4 biological replicates). (F) Flow cytometry analysis of propidium iodide (PI) stained trophozoites preconditioned with DMEM/F-12, DMEM/F-12+FBS, or TYDK, and stained positive control cells (Triton x-100 treated trophozoites) (n = 3 biological replicates). The upper right panel illustrates quantification of the fraction of stained cells in the positive gate. The lower right panel shows the quantification of the median PI fluorescent intensity of negative gated cells. Statistical significance was determined using Welchs’ t-test with Holm-type corrections for multiple testing. *p < 0.05, **p < 0.01, ***p < 0.001, ****p < 0.0001, ns = not significant. *G*.*i*., *Giardia intestinalis*; TYDK, *G*. *intestinalis* growth media.
(TIF)

S4 Fig Cytokine protein concentrations in culture media of IECs in the apical compartment. ELISA measurements of CXCL1 and CXCL8 protein concentrations in culture media supernatants of the apical compartment of uninfected IEC monolayers and IEC monolayers infected with DMEM/F-12+FBS preconditioned trophozoites, DMEM/F-12 preconditioned trophozoites, or lysed trophozoites at MOI1.2 (3 h and 6 h p.i.) (n = 3 biological replicates). The black dotted lines indicate the detection limit (concentration of lowest standard sample). Statistical significance was determined using a one-way analysis of variance (ANOVA), followed by Bonferroni multiple comparison test. *p < 0.05, **p < 0.01, ***p < 0.001, ns = not significant. *G*.*i*., *Giardia intestinalis*
(TIF)

S5 Fig Immune responses of intestinal epithelial cells are influenced by trophozoites’ fitness. (A) qPCR of cytokine mRNA expression levels in IECs during infection with DMEM/F-12+FBS preconditioned trophozoites, heat-inactivated (HI) trophozoites, or lysed trophozoites (1 h p.i.) (n = 3 biological replicates, ± SD). Fold change values of all samples were calculated by comparing to the 1 h uninfected control. Statistical significance was determined using Welchs’ t-test with Holm-type corrections for multiple testing. *p < 0.05, **p < 0.01, ***p < 0.001, ns = not significant. (B) *G*. *intestinalis* trophozoite attachment to IECs during DMEM/F-12+FBS preconditioned trophozoite infection, or DMEM/F-12+FBS trophozoite plus lysed trophozoite co-infection. Note that data presented in this figure and in Fig 3B and derive from the same infection experiment and some sample groups appear in both plots. *G*.*i*., *Giardia intestinalis*.
(TIF)

S6 Fig RNA sequencing of in intestinal epithelial cell infections with “fit” or “non-fit” *Giardia intestinalis* trophozoites. (A) Read counts mapped to either the human or the *G*. *intestinalis* reference genome. (B) Plot of the principle component analysis (PCA) illustrating PC1 and PC2 of the top 100 human variant genes. (n = 3 biological replicates). (C) Venn diagram of differentially expressed human IEC genes between “fit” (DMEM/F-12+FBS) or “non-fit” (DMEM/F-12) *G*. *intestinalis* infection samples and their respective controls at 1 h or 3 h p.i.. *G*.*i*, *Giardia intestinalis*
(TIF)

S7 Fig Highly up- and down-regulated host genes. Heatmap of the log2 fold change values of the top 20 upregulated **(A)** and downregulated **(B)** IEC DEGs during “fit” (DMEM/F-12+FBS) and “non-fit” (DMEM/F-12) trophozoite infection, at each of the infection time points (1 h and 3 h). Only significant values are shown.
(TIF)

S8 Fig Enriched gene ontology and reactome pathway terms in differentially expressed host genes during “fit” and “non-fit” trophozoite infection. Dot plot showing gene ontology (GO) and reactome pathway terms enriched in host differentially expressed genes (DEGs) related to apical junction, cell death, immune response, metabolism, oxidative stress, protein stability/localization, signalling, as well as transcription and translation. Infection condition and infection time points are indicated at the x-axis. Circle size and colour indicate the number of DEGs and their significance (adjusted p-value), respectively. BP, Biological Processes; MF, Molecular Function; up, upregulated DEGs; down, downregulated DEGs.
(TIF)

S9 Fig RNA sequencing of “fit” or “non-fit” *Giardia intestinalis* trophozoites after preconditioning and during in intestinal epithelial cell infection. (A) Volcano plots showing differentially expressed *G*. *intestinalis* genes between “fit” (DMEM/F-12+FBS) or “non-fit” (DMEM/F-12) preconditioned trophozoite infections and the respective 0h inoculum at 1h and 3h p.i. (n = 3 biological replicates). Each dot represents a DEG and are coloured according to expression fold change and false discovery rate (FDR) (grey, FDR > 0.05; dark blue FDR < 0.05 and log2FC < -1; light blue FDR < 0.05 and log2FC < 0; dark red FDR < 0.05 and log2FC > 1; light red FDR < 0.05 and log2FC > 0). (B) Venn diagram of *G*. *intestinalis* DEGs between “fit” (DMEM/F-12+FBS) or “non-fit” (DMEM/F-12) infection samples and their respective 0 h inoculum at 1 h and 3 h p.i.. (C) Heatmap showing the expression of *G*. *intestinalis* genes differentially expressed between “fit” (DMEM/F-12+FBS) and “non-fit” (DMEM/F-12) inoculum samples at 0h. Genes annotated in grey are higher expressed in “non-fit” trophozoites and genes annotated in yellow are higher expressed in “fit” trophozoites. Expression values are z-score transformed transcript counts. (D) VSP (variant-specific surface protein) gene expression of the 0 h inoculum samples are plotted as log 10 GeTMM [91] normalized counts. Colours indicate if VSPs are differentially expressed between “fit” (DMEM/F-12+FBS) and “non-fit” (DMEM/F-12) inoculum samples.
(TIF)

S1 Table Differentially expressed human genes. (XLSX)

S2 Table Uniquely differentially expressed human genes in the different infection conditions. (XLSX)

S3 Table GO term and GSEA enrichment of differentially expressed human genes. (XLSX)

S4 Table Differentially expressed *G*. *intestinalis* genes. (XLSX)

S5 Table qPCR primer sequences. (TIF)

S1 Video Live imaging of human enteroid-derived 2D monolayer infections with *Giardia intestinalis* trophozoites. The movie shows a real-time example of *G*. *intestinalis*-mNeonGreen trophozoites swimming and attaching to a PET-transwell enteroid monolayer with DMEM/F-12 as apical infection media and ENR as the basal compartment media. *G*. *intestinalis* trophozoites were preconditioned with DMEM+FBS. Scale bar = 100 μm. Time post infection is indicated as hours: minutes: seconds.
(MOV)
