# Supplementary figures and images for: Trophozoite fitness dictates the intestinal epithelial cell response to Giardia intestinalis infection
Source: PLoS Pathog. 2023 May 4;19(5):e1011372. doi: 10.1371/journal.ppat.1011372 (PMC10187934; doi:10.1371/journal.ppat.1011372)

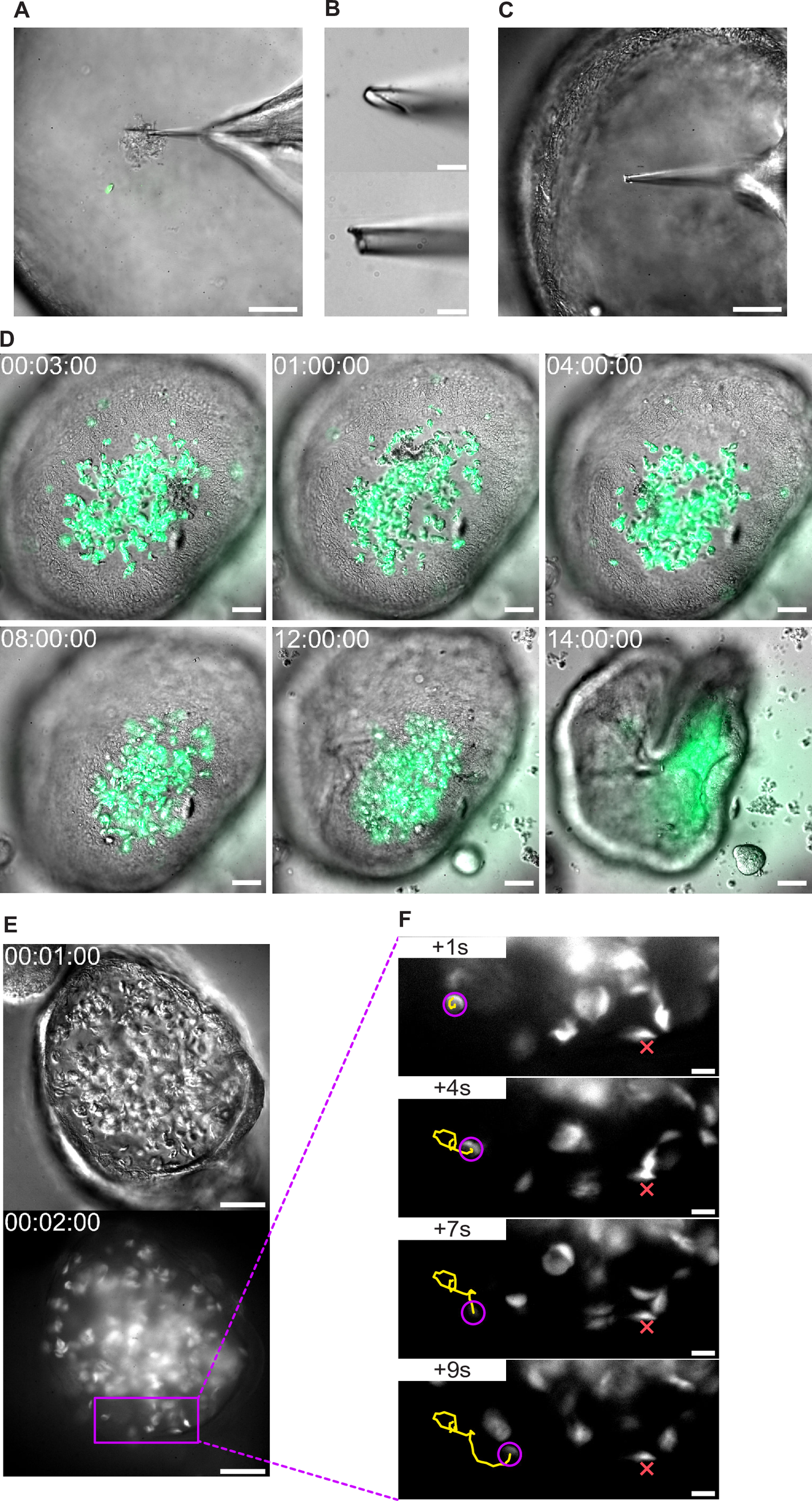

Supplement: S1 Fig — (A) G. intestinalis microinjection with a non-optimised microinjection needle (<7μm tip diameter) caused trophozoites to rupture and clog the needle. Scale bar = 50 μm. (B) Tips of optimised G. intestinalis microinjection needles with wider openings (7–9 μm diameter). Scale bar = 10 μm. (C) Microinjection of a 3D enteroid with an optimised G. intestinalis needle that is clogged by G. intestinalis trophozoites. Scale bar = 50 μm. (D) Representative 3D enteroid successfully microinjected with G. intestinalis-mNeonGreen trophozoites imaged over the course of the infection until the enteroid collapsed. Time post infection is indicated as hours: minutes: seconds. Scale bar = 50 μm. (E) Microinjection of G. intestinalis-mNeonGreen trophozoites into 3D enteroids visualized by DIC and fluorescence microscopy imaging (upper and lower panel, respectively). Time post infection is indicated as hours: minutes: seconds. (F) Time-lapse microscopy of trophozoites in the mNeonGreen fluorescence channel. Manual tracking of a single trophozoite swimming atop the IEC surface inside the enteroid. The tracked trophozoite is indicated by magenta circles and the track by yellow lines. Representative attached trophozoite is indicated by a red cross. Scale bar = 10 μm. (TIF) [file ppat.1011372.s001.tif]

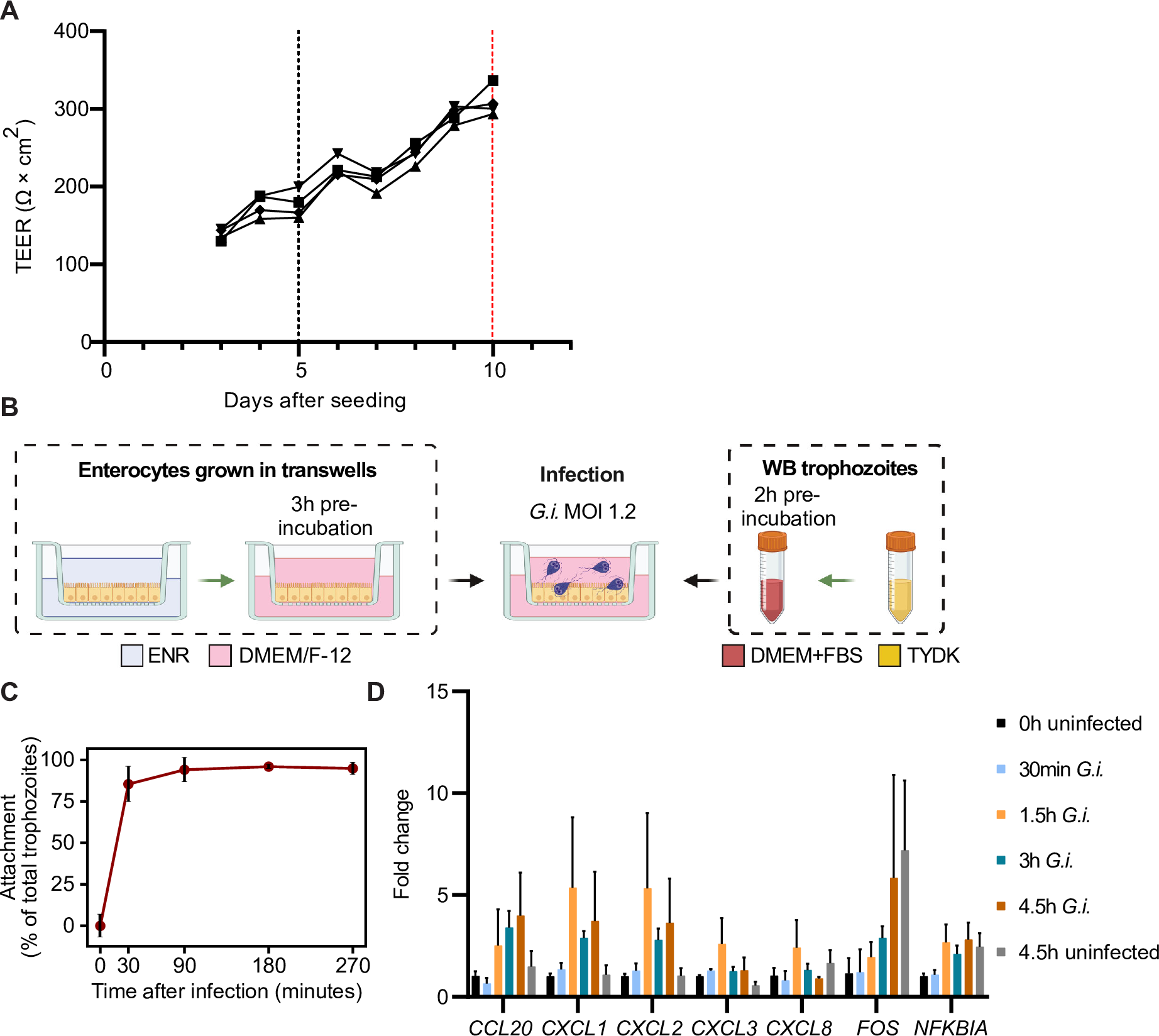

Supplement: S2 Fig — (A) Human jejunal enteroid IEC monolayers were grown on PET-transwells and TEER measurements were performed to verify an increase in confluency and barrier integrity over time (n = 4 biological replicates). The black dotted line indicates the medium change to differentiation media (ENR) at day 5 after seeding and the red dotted line indicates the day of infection at day 10 after seeding. (B) Schematic representation showing the jejunal enteroid-derived IEC monolayer infection model, using only DMEM/F-12 as the infection media. Created with BioRender.com. (C) G. intestinalis trophozoite attachment to IECs over the course of the monolayer infection. (D) qPCR of host cell response genes at 30 min, 1.5 h, 3 h and 4.5 h post G. intestinalis infection, including 0h and 4.5h negative controls (n = 3 biological replicates ± SD). Fold change values of all samples were calculated by comparing to the 0 h uninfected control. G.i., Giardia intestinalis; ENR, enterocyte differentiation media; TYDK, G. intestinalis growth media. (TIF) [file ppat.1011372.s002.tif]

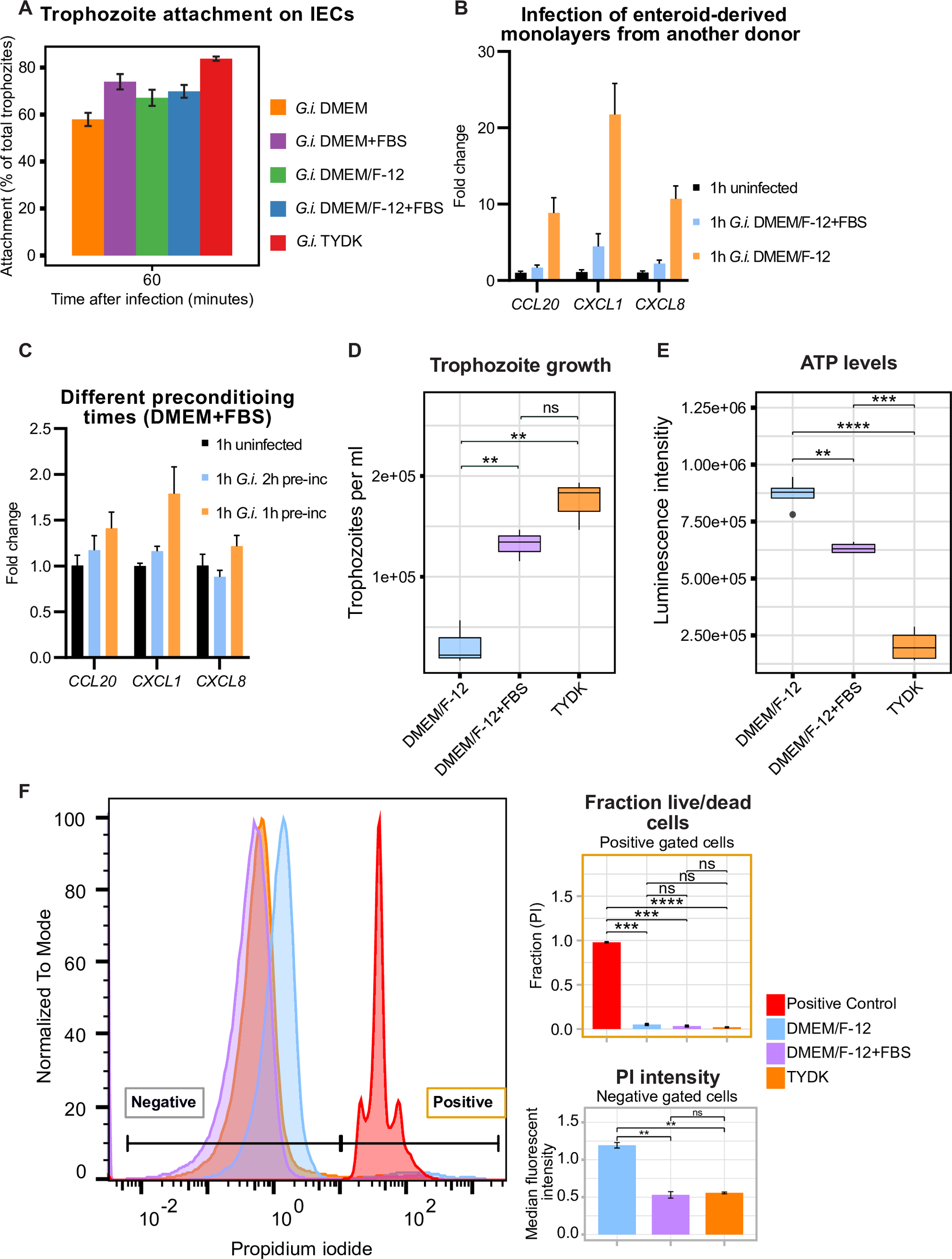

Supplement: S3 Fig — (A) G. intestinalis trophozoite attachment to IECs after 1h of infection. Trophozoites were preconditioned with DMEM+FBS, DMEM, DMEM/F-12+FBS, DMEM/F-12, or TYDK for 1h before the infection. (B) Preconditioned trophozoite (DMEM/F-12+FBS or DMEM/F-12) infections (MOI1.2) of enteroid-derived IEC monolayers established from another human donor culture. qPCR of chemokine mRNA expression levels at 1 h post G. intestinalis infection (n = 4 biological replicates, except n = 5 for 1 h G. intestinalis DMEM/F-12 preconditioning infection ± SD). Fold change values of all samples were calculated by comparing to the 1 h uninfected control. (C) qPCR of chemokine mRNA expression levels in IECs infected for 1 h with G. intestinalis trophozoites preconditioned with DMEM+FBS either for 1 h, or 2 h (n = 3 biological replicates ± SD). Fold change values of all samples were calculated by comparing to the 1 h uninfected control. (D) Trophozoite growth after 1 h of preconditioning in DMEM/F-12, DMEM/F-12+FBS or TYDK (n = 3 biological replicates). (E) Intracellular ATP levels of trophozoites after 1h of preconditioning measured with CellTiter-Glo cell viability assay (n = 4 biological replicates). (F) Flow cytometry analysis of propidium iodide (PI) stained trophozoites preconditioned with DMEM/F-12, DMEM/F-12+FBS, or TYDK, and stained positive control cells (Triton x-100 treated trophozoites) (n = 3 biological replicates). The upper right panel illustrates quantification of the fraction of stained cells in the positive gate. The lower right panel shows the quantification of the median PI fluorescent intensity of negative gated cells. Statistical significance was determined using Welchs’ t-test with Holm-type corrections for multiple testing. *p < 0.05, **p < 0.01, ***p < 0.001, ****p < 0.0001, ns = not significant. G.i., Giardia intestinalis; TYDK, G. intestinalis growth media. (TIF) [file ppat.1011372.s003.tif]

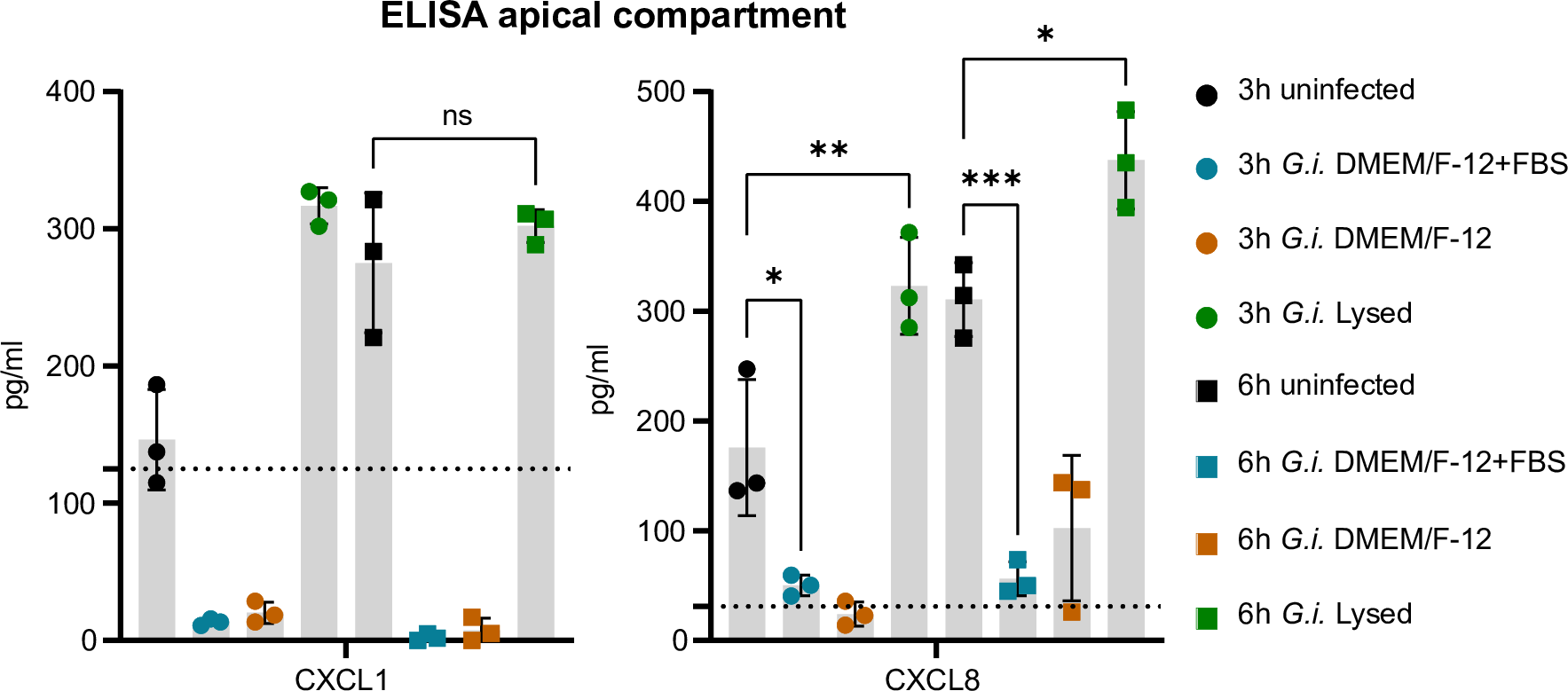

Supplement: S4 Fig — ELISA measurements of CXCL1 and CXCL8 protein concentrations in culture media supernatants of the apical compartment of uninfected IEC monolayers and IEC monolayers infected with DMEM/F-12+FBS preconditioned trophozoites, DMEM/F-12 preconditioned trophozoites, or lysed trophozoites at MOI1.2 (3 h and 6 h p.i.) (n = 3 biological replicates). The black dotted lines indicate the detection limit (concentration of lowest standard sample). Statistical significance was determined using a one-way analysis of variance (ANOVA), followed by Bonferroni multiple comparison test. *p < 0.05, **p < 0.01, ***p < 0.001, ns = not significant. G.i., Giardia intestinalis (TIF) [file ppat.1011372.s004.tif]

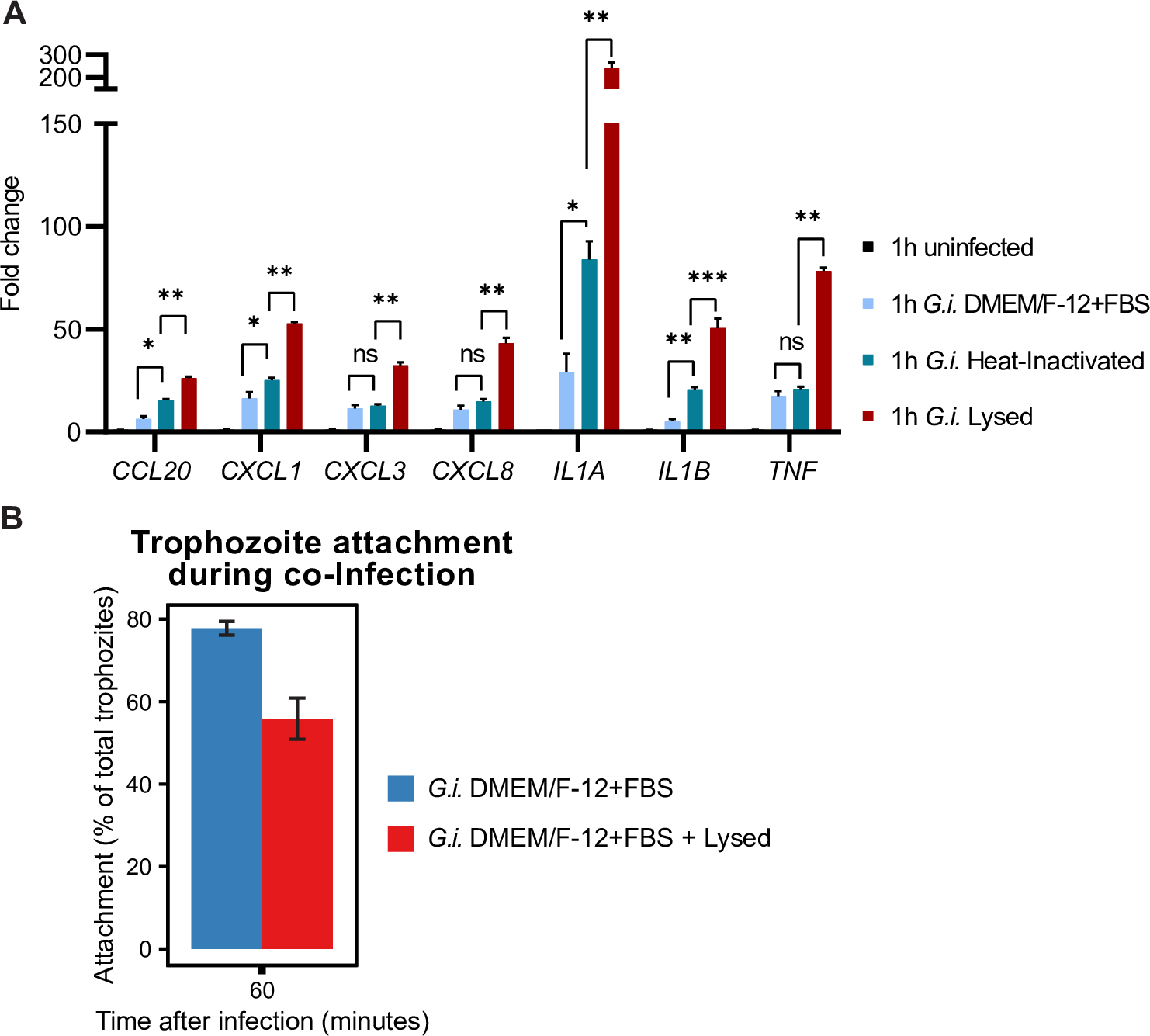

Supplement: S5 Fig — (A) qPCR of cytokine mRNA expression levels in IECs during infection with DMEM/F-12+FBS preconditioned trophozoites, heat-inactivated (HI) trophozoites, or lysed trophozoites (1 h p.i.) (n = 3 biological replicates, ± SD). Fold change values of all samples were calculated by comparing to the 1 h uninfected control. Statistical significance was determined using Welchs’ t-test with Holm-type corrections for multiple testing. *p < 0.05, **p < 0.01, ***p < 0.001, ns = not significant. (B) G. intestinalis trophozoite attachment to IECs during DMEM/F-12+FBS preconditioned trophozoite infection, or DMEM/F-12+FBS trophozoite plus lysed trophozoite co-infection. Note that data presented in this figure and in Fig 3B and derive from the same infection experiment and some sample groups appear in both plots. G.i., Giardia intestinalis. (TIF) [file ppat.1011372.s005.tif]

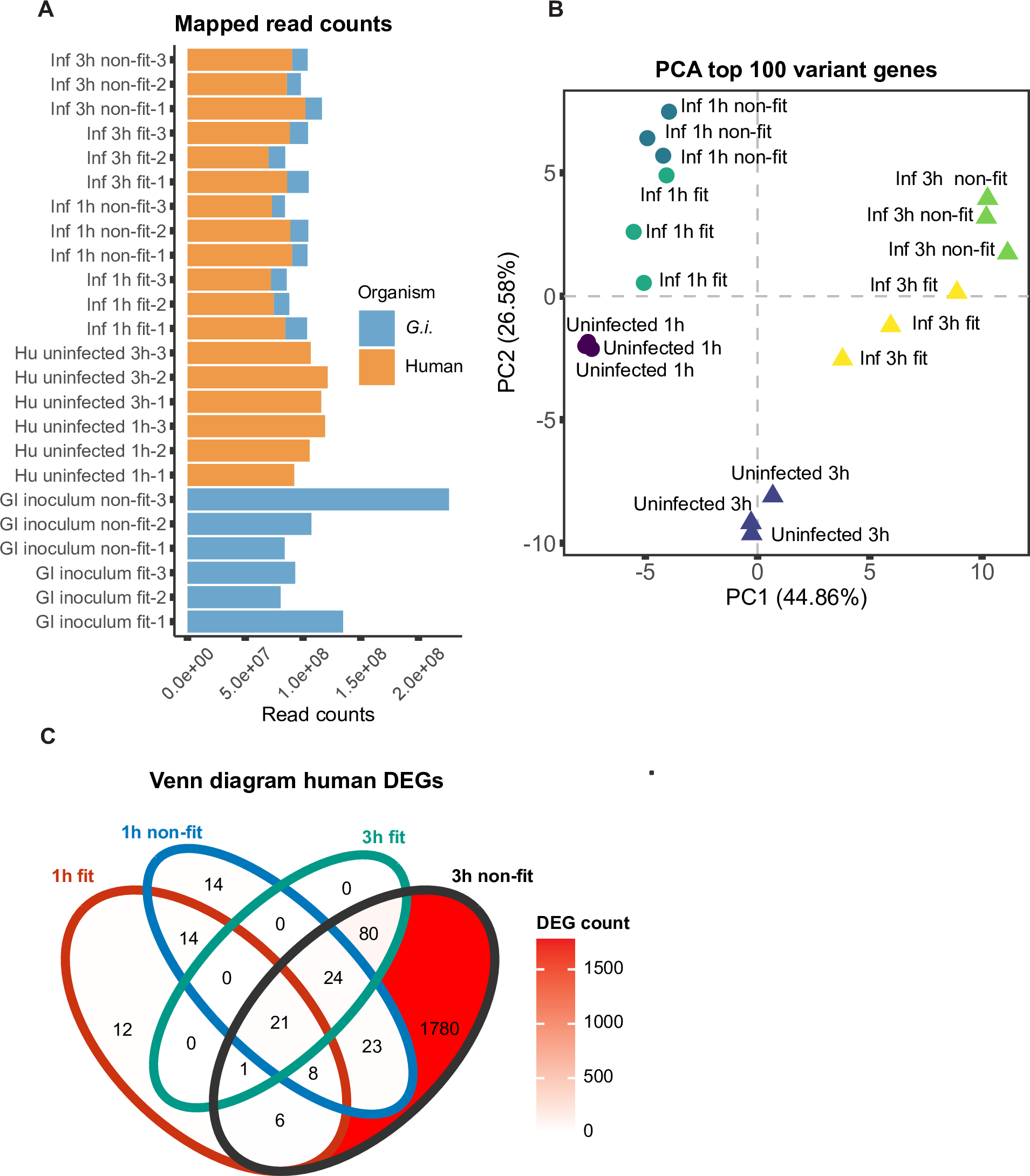

Supplement: S6 Fig — (A) Read counts mapped to either the human or the G. intestinalis reference genome. (B) Plot of the principle component analysis (PCA) illustrating PC1 and PC2 of the top 100 human variant genes. (n = 3 biological replicates). (C) Venn diagram of differentially expressed human IEC genes between “fit” (DMEM/F-12+FBS) or “non-fit” (DMEM/F-12) G. intestinalis infection samples and their respective controls at 1 h or 3 h p.i.. G.i, Giardia intestinalis (TIF) [file ppat.1011372.s006.tif]

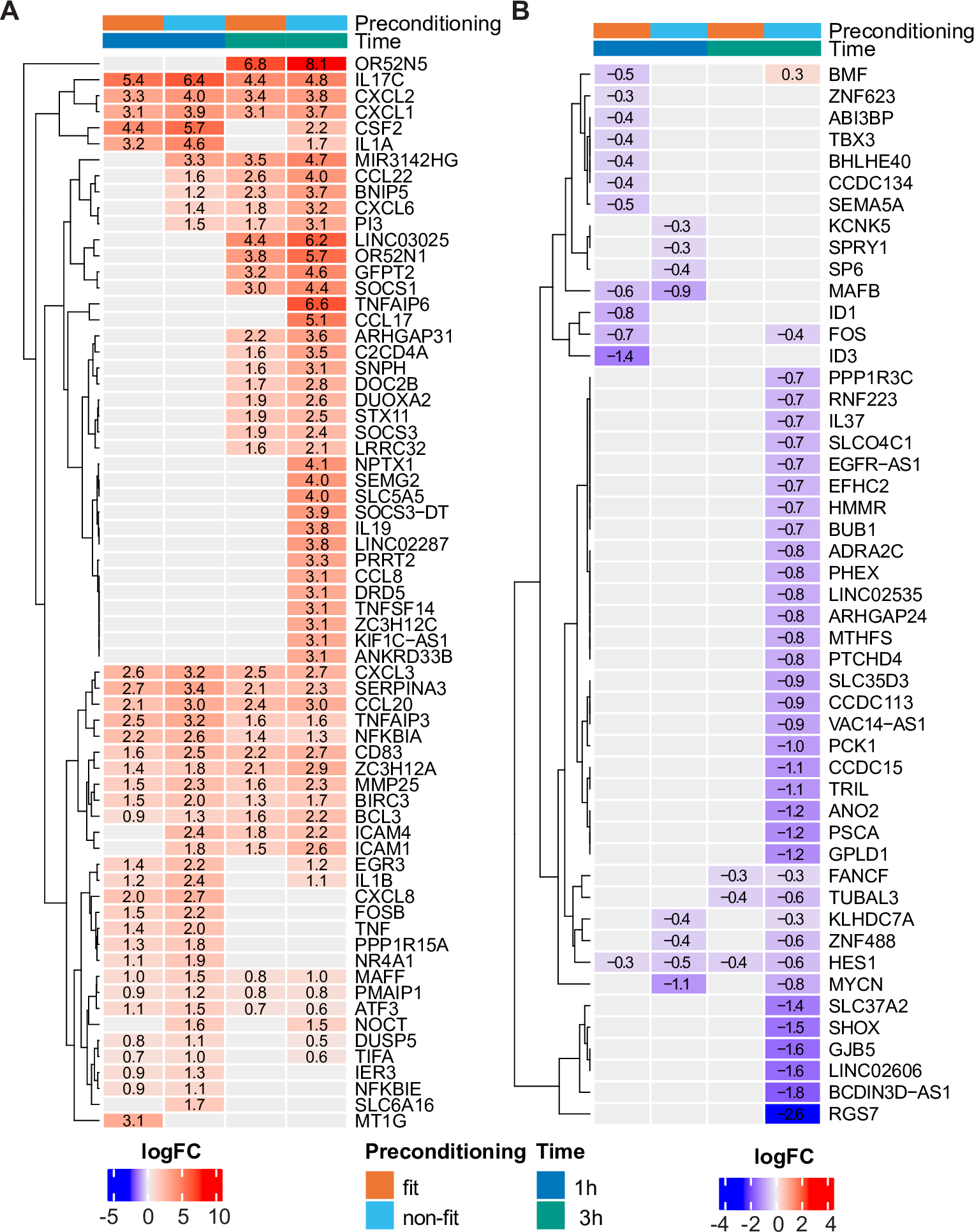

Supplement: S7 Fig — Heatmap of the log2 fold change values of the top 20 upregulated (A) and downregulated (B) IEC DEGs during “fit” (DMEM/F-12+FBS) and “non-fit” (DMEM/F-12) trophozoite infection, at each of the infection time points (1 h and 3 h). Only significant values are shown. (TIF) [file ppat.1011372.s007.tif]

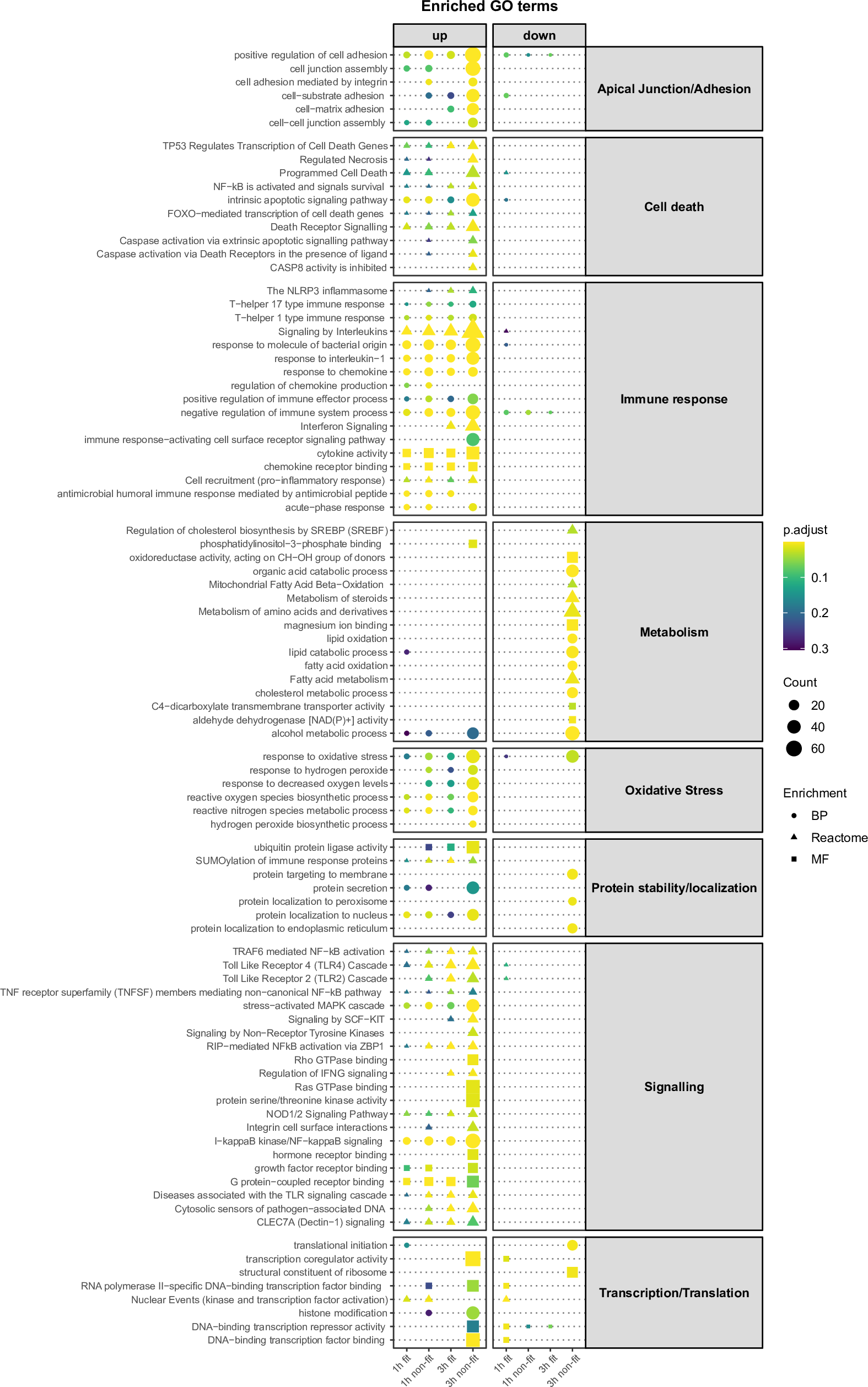

Supplement: S8 Fig — Dot plot showing gene ontology (GO) and reactome pathway terms enriched in host differentially expressed genes (DEGs) related to apical junction, cell death, immune response, metabolism, oxidative stress, protein stability/localization, signalling, as well as transcription and translation. Infection condition and infection time points are indicated at the x-axis. Circle size and colour indicate the number of DEGs and their significance (adjusted p-value), respectively. BP, Biological Processes; MF, Molecular Function; up, upregulated DEGs; down, downregulated DEGs. (TIF) [file ppat.1011372.s008.tif]

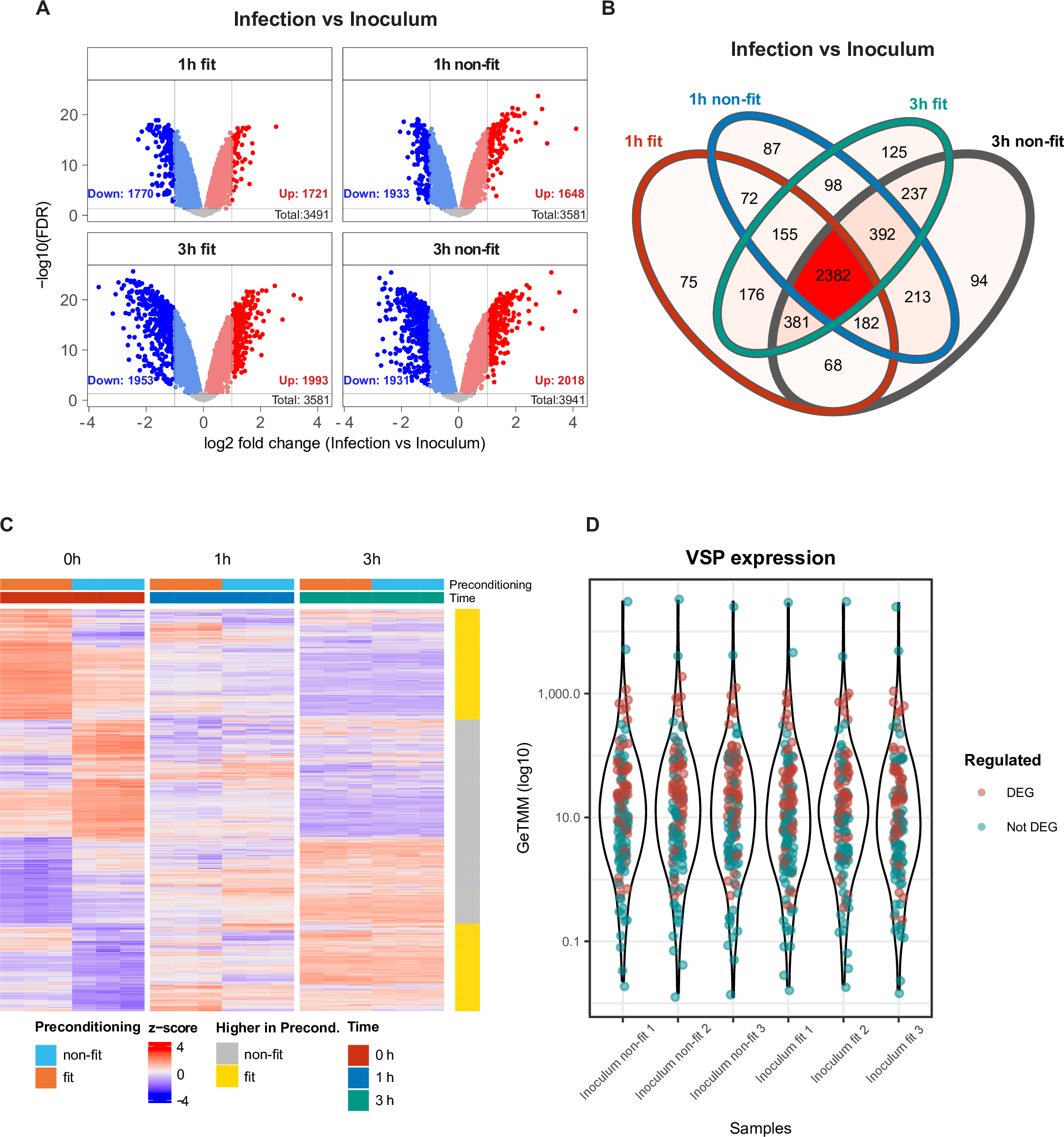

Supplement: S9 Fig — (A) Volcano plots showing differentially expressed G. intestinalis genes between “fit” (DMEM/F-12+FBS) or “non-fit” (DMEM/F-12) preconditioned trophozoite infections and the respective 0h inoculum at 1h and 3h p.i. (n = 3 biological replicates). Each dot represents a DEG and are coloured according to expression fold change and false discovery rate (FDR) (grey, FDR > 0.05; dark blue FDR < 0.05 and log2FC < -1; light blue FDR < 0.05 and log2FC < 0; dark red FDR < 0.05 and log2FC > 1; light red FDR < 0.05 and log2FC > 0). (B) Venn diagram of G. intestinalis DEGs between “fit” (DMEM/F-12+FBS) or “non-fit” (DMEM/F-12) infection samples and their respective 0 h inoculum at 1 h and 3 h p.i.. (C) Heatmap showing the expression of G. intestinalis genes differentially expressed between “fit” (DMEM/F-12+FBS) and “non-fit” (DMEM/F-12) inoculum samples at 0h. Genes annotated in grey are higher expressed in “non-fit” trophozoites and genes annotated in yellow are higher expressed in “fit” trophozoites. Expression values are z-score transformed transcript counts. (D) VSP (variant-specific surface protein) gene expression of the 0 h inoculum samples are plotted as log 10 GeTMM [91] normalized counts. Colours indicate if VSPs are differentially expressed between “fit” (DMEM/F-12+FBS) and “non-fit” (DMEM/F-12) inoculum samples. (TIF) [file ppat.1011372.s009.tif]

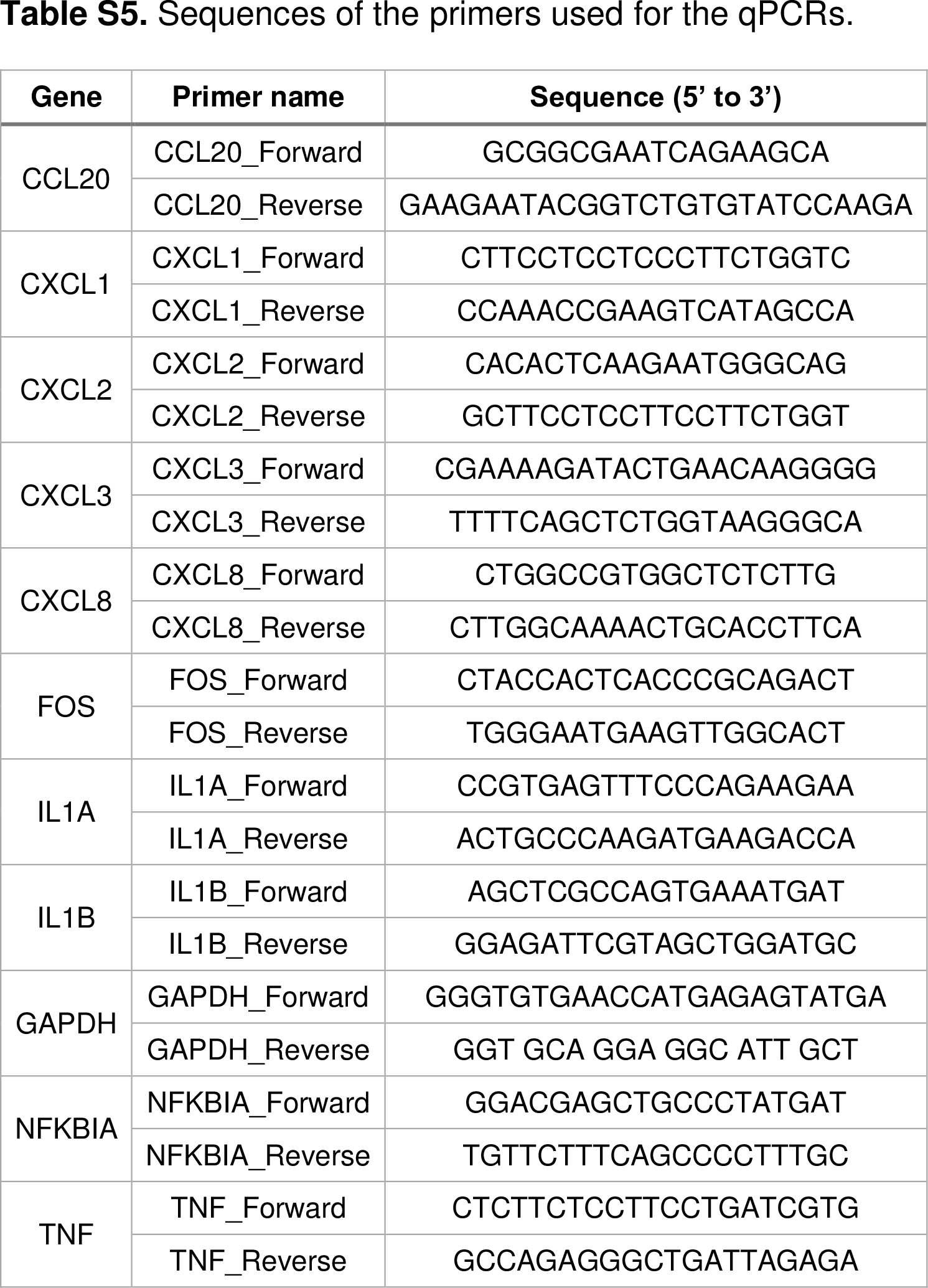

Supplement: S5 Table — (TIF) [file ppat.1011372.s014.tif]
